# A Meta-Analytical Way of Systematizing the Use of Hyaluronan Gels for the Relief of Osteoarthritis, Compared with New Treatment Alternatives

**DOI:** 10.3390/gels10070481

**Published:** 2024-07-20

**Authors:** Tudor Pinteala, Stelian Sergiu Maier, Norin Forna, Liliana Savin, Mihnea Theodor Sirbu, Dragos Cristian Popescu, Viorel Dan Cionca, Dan Constantin Putineanu, Paul Dan Sirbu

**Affiliations:** 1 Department of Orthopedics and Traumatology, Faculty of Medicine, “Grigore T. Popa” University of Medicine and Pharmacy, 700115 Iasi, Romania; tudor_pinteala@umfiasi.ro (T.P.); norin.forna@umfiasi.ro (N.F.); liliana.savin@umfiasi.ro (L.S.); mihnea-theodor.pd.sirbu@students.umfiasi.ro (M.T.S.); dragos.popescu@umfiasi.ro (D.C.P.); paul.sirbu@umfiasi.ro (P.D.S.); 2 Department Orthopedics and Traumatology, Clinical Rehabilitation Hospital, 700661 Iasi, Romania; 3 Department of Chemical Engineering, Faculty of Industrial Design and Business Management, “Gheorghe Asachi” Technical University of Iasi, 73 Mangeron Blvd., 700050 Iasi, Romania; 4 Department of Orthopaedics and Traumatology, “Sf. Spiridon” Emergency Universitary Hospital, 700115 Iasi, Romania; dan.cionca@spitalspiridon.ro; 5 Orthopedic Surgery Department, Cliniques Universitaires Saint-Luc, 1200 Brussels, Belgium; dan.putineanu@saintluc.uclouvain.be

**Keywords:** hyaluronic acid, viscosupplementation, platelet-rich plasma, osteoarthritis, meta-analysis, meta-regression, reproducible research

## Abstract

Hyaluronic acid, in the form of a gel or viscoelastic colloidal solution, is currently used for the viscosupplementation of joints affected by osteoarthritis, but its effectiveness is under debate in relation to newer alternatives. Based on meta-analytical arguments, the present article reinforces the opinion that there are still no decisive arguments for its complete replacement but for its use adapted to the peculiarities of the disease manifestation and of the patients. A “broad” comparison is first made with almost all alternatives studied in the last decade, and then a meta-regression study is performed to compare and predict the effect size induced by viscosupplementation therapy and its main challenger of clinical interest, the platelet-rich plasma treatment. If they are computerized, the developed models can represent tools for clinicians in determining the appropriateness of the option or not for viscosupplementation in a manner adapted to the pain felt by the patients, to their age, or to other clinical circumstances. The models were generated using algorithms implemented in the R language and assembled in different R packages. All primary data and necessary R scripts are provided in accordance with the philosophy of reproducible research. Finally, we adhere in a documented way to the opinion that HA-based products, currently under circumspection, are still clinically useful.

## 1. Introduction

Osteoarthritis (OA) is a progressive degenerative disease of multifactorial etiology characterized by joint stiffness, swelling, pain, and loss of movement. These symptoms arise from the loss of articular cartilage and periarticular bone remodeling. It is estimated that OA affects almost 500 million people worldwide (7% of the world population, approximately half of the world’s population over the age of 65) [1].

**Figure 1:**
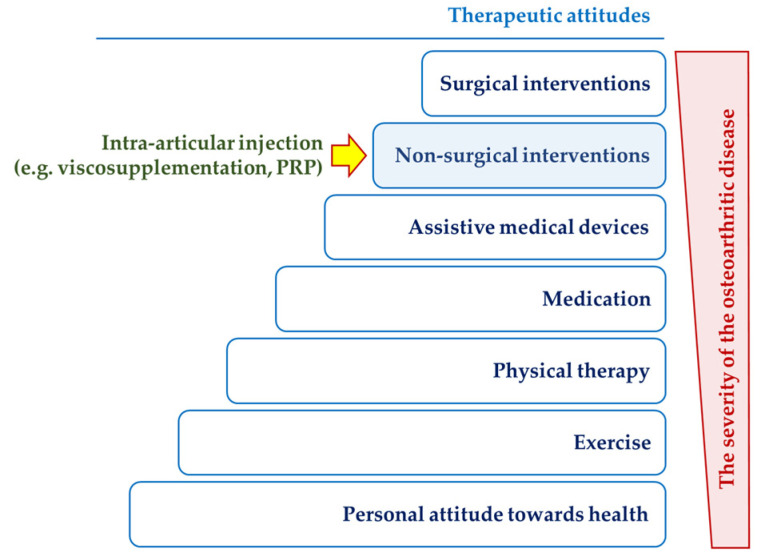
The position of injectable treatments in the hierarchy of OA treatment options.

The knee is typically the joint most affected by osteoarthritis, followed by the hip, hand, spine, and feet. This high prevalence of knee osteoarthritis (KOA) is attributed to several factors, such as population aging, rising obesity rates, demographic growth, gender, diet, and an increase in joint injuries. Among them, age often emerges as the most significant determinant of knee KOA onset and severity [2]. The primary therapeutic goals for patients suffering from KOA are to reduce pain, improve mobility, promote cartilage regeneration, and restore overall function [3]. In cases of advanced knee osteoarthritis, total joint arthroplasty is the recommended treatment option; however, in the early stages of KOA, a range of therapeutical options exist that can effectively alleviate the symptoms caused by the disease. These options include intra-articular administration of medications or oral painkillers such as paracetamol, opioids, and non-steroidal anti-inflammatory drugs (NSAIDs). However, due to their high toxicity and low tolerance after long periods, intraarticular (IA) treatments are often preferred by clinicians and patients [4]. A hierarchy of general OA treatment methods is shown in Figure 1. Therapeutic attitudes are adopted depending on the severity of the disease. Intra-articular injections belong to the non-surgical methods and are applied, possibly combined with other methods (most often with *per os* medication), in forms resistant to other treatments.

It is already accepted that once initiated, there is no clinical intervention able to completely stop the advance or irreversibly cure OA [5,6]. One conservative treatment option for reducing patients’ symptoms caused by KOA consists of minimally invasive intra-articular injections. Among the many variants tested over the years [7], three types are currently applied: (i) injections for short-term pain relief (by using analgesics and anti-inflammatory drugs), (ii) injections for long-term suffering alleviation and/or mobility rehabilitation (that make use of NSAIDs, hyaluronic acid (HA), platelet-rich plasma (PRP), or various mixtures thereof with or without other small molecule drugs), and (iii) injections for the robust fight against inflammation through immunomodulation (by means of cortisol mimicking drugs, mesenchymal stem cells, ozone, etc.).

Hyaluronic acid is one of the most widely applicable products, both for reducing the symptoms of OA and for delaying the structural deterioration of the joints. Once introduced into the synovial fluid, it mainly plays the role of joint lubricant and shock absorber. Since HA is naturally present in both healthy and injured articulations, the medical act of adding it by injection is called viscosupplementation of the synovial fluid.

The synovial fluid content of the joints varies depending on their size, the mechanical effort to which they are usually subjected, and the physiological or pathological state in which the joint functions. As a general rule, joint diseases induce an increase in the volume of synovial fluid but lead to a decrease in its rheological characteristics and, therefore, to a significant loss of its lubricating capacity. Properly performed physical effort works in the opposite direction. In the healthy knee joint, the physiological volume of the synovial fluid is 6.7 ± 2.3 mL (with a median of 7.2 mL), but it reaches 13.6 ± 7.4 mL in latent gonarthrosis and 24.2 ± 16.3 mL in active gonarthrosis [8,9]. The concentration of HA in healthy joints is about 1 to 4 mg/mL and decreases to 0.1 to 1.3 mg/mL in the arthritic ones [10]. The molecular weight of HA in synovial fluid varies between 2 and 12 MDa (6.5 and 10.9 MDa in the knee joint) [10] and decreases to half of the value in OA [11]. The zero shear viscosity of the synovial fluid drops dramatically in osteoarthritis, from 10–34 Pa‧s to 0.1–1.0 Pa‧s [12,13], while the rate index (the dependency of shear-thinning viscosity on shear rate) slightly decreases, from 0.75 in healthy condition to 0.6 in OA [12].

As a symptomatic treatment of OA, viscosupplementation aims to restore the rheological behavior of synovial fluid by the controlled addition of exogenous HA. Furthermore, it concludes by (i) balancing the volume of synovial fluid in the affected joint, (ii) increasing synovial fluid viscoelasticity, which was diminished in OA, (iii) stimulating intra-articular production and turnover of HA into the synovial fibroblasts, (iv) reducing inflammation by immunologic modulation, and (v) delaying joint degradation by decreasing matrix metalloproteinase biosynthesis [14,15,16,17]. The multiple beneficial effects of intra-articular HA injections have accredited viscosupplementation as a versatile, highly effective clinical option in the management of osteoarticular pain and disease [18]. The aforementioned effects, both complex and dynamic, have determined that viscosupplementation has remained an easy and cost-effective alternative throughout its more than 50-year history as a medical treatment [19]. Although in these decades, a unique mechanism of action of HA in OA has not been established, in clinical terms, the main contributor to the relief of suffering is considered to be the physical supplementation or even the total replacement of the deficient synovial fluid with a sterile HA-based hydrogel, either in its native state or chemically functionalized. In practice, supplementation is carried out after removing at least a fraction of the impaired synovial liquid so that the reconstitution of its volume and physical–chemical and rheological characteristics can reach values as close as possible to physiological ones. All the other positive effects of the presence of additional HA in the joint are the same as those provided by the HA naturally biosynthesized in the synovial sheath. Therefore, the mechanism of reduction in osteoarticular pain by viscosupplementation is essentially the same as that which is induced by the healthy synovial liquid during joint self-repair. In this context, viscosupplementation offers “the most natural way possible” to alleviate OA suffering.

To be used for viscosupplementation, HA is produced by controlled biosynthesis in recombinant bacteria cultures [20,21], then formulated [22,23], derivatized [24,25,26], and possibly crosslinked [27,28] to increase its resistance against intra-articular enzymatic degradation. As a result, HA sterile assortments having molecular weights of 20 to 1000 kDa (rarely up to 5000 kDa) and stable viscosity values of 0.1 to 1.1 Pa‧s [29] are available for clinical use. Commercial products incorporate HA gels (or elasto-viscous colloidal solutions) of 10 to 20 mg/mL, buffered to neutral pH and adjusted with salt or saccharides to approximately 290 to 335 mOsm/kg, packaged in prefilled syringes with volumes of 1 to 4 mL per dose. The dosage of such a product is determined by the physician depending on the degree of damage to the joint and on the joint size (the volume of synovial fluid it naturally contains). The intra-articular dosage of HA gels aims to increase the volume of synovial fluid to the physiological level or even to replace it completely, once or periodically.

Besides the usual additions of mannitol and niacinamide made to adjust osmolarity and improve stability, respectively, the limited supplemental formulation may be considered for HA-based products [30]. Even if their therapeutic or theranostic validity has not yet been certified by clinical studies, for a series of pharmacologically active compounds, in vitro tests have been performed to evaluate their physicochemical effect on the stability of newly formulated products. Tested compounds include tribological modulators [31], stabilizers against intra-articular hyaluronidase [32], analgesics [33], and iodinated contrast agents (for arthrography) [34]. They have been proposed (but only a few have been approved by regulatory agencies) as components in the formulas of so-called active viscosupplementing agents [35].

In medical practice, the main challenge of HA is the extemporaneously prepared autologous PRP [36]. The latter has, in most cases, the characteristics of a colloidal suspension or of a very loose physical gel, with apparent viscosities of the order of 0.01 Pa‧s [37]. It can be transformed into a platelet-rich plasma gel matrix (PRP-GM) by plasmatic protein polymerization [38] in order to be directly administered or to be further synergistically formulated along with HA as complex structured gels applicable for viscosupplementation [39,40] prepared according to the Cellular Matrix^®^ technology (Regen Lab, Le Mont-sur-Lausanne, Switzerland) [41,42]. The amount of PRP per administered dose is usually adjusted to conform to the volume per dose of commercial HA products (1 to 4 mL). Variants of PRP derivatives have also been clinically tested, like the plasma rich in growth factors (PRGF) [43] or platelet-rich plasma-derived growth factor (PGRF) [44], but their extemporaneous preparation is still too difficult to be widely applied.

One of the obstacles to evaluating the effectiveness of treatments in OA derives from the subjectivity of reporting the pain felt by the patient at the level of the joint before and after the treatment. Even if objective assessment of the stage and evolution of OA is possible (for example, by radiological imaging), it is not always feasible, mainly because of the costs involved in the instrumental investigations applied to large numbers of patients during clinical trials. Most clinical investigations still use subjective scales to assess joint pain and/or discomfort based on questionnaires completed by patients, according to the technique of self-reporting outcomes [45]. For this reason, the comparison and systematization of clinical studies in OA can only be made through meta-analyses and meta-regression, even those affected by significant variability/heterogeneity/uncertainty when applied to pre- and post-treatment pain scores. Usually, the evaluating scales are highly peculiar, and no direct conversion between their scores is available or even possible. However, this deficiency can be overcome by calculating the size effect of the treatment outcome, which is a dimensionless numerical value, thus being able to be the subject of statistical (and possibly narrative) comparison. The approaches based on meta-analysis techniques (which represent a framework for the statistical treatment of distinctive sources of information in order to substantiate an assessment or a decision, here of clinical value, usually by combining the results reported in several studies) are largely reported in order to (periodically) assess the changes in the acceptance of OA treatment options from the perspective of patients.

Score-based qualitative evaluation of OA status and stage has been frequently criticized, and several improvements have been proposed [46,47], especially to try to avoid the evaluation of unsimilar or divergent aspects of joint pain when distinctive scales and subscales are considered. Besides the newest scale, the Osteoarthritis Symptom Inventory Scale (OASIS), older ones are still used, like WOMAC (Western Ontario and McMaster Universities), VAS (Visual Analogue Scale), KOOS (Knee Injury and Osteoarthritis Outcome Score), or IKDC (International Knee Documentation Committee). The most perturbing issue when reports based on distinctive pain (sub)scales are used for meta-analysis consists of the significant differences in sensitivity of those (sub)scales in pain evaluation. Such differences increase the incertitude of the meta-analysis conclusions and induce unjustified and improper biases between the compared clinical studies. Fortunately, modern meta-analysis methods are capable of highlighting (but not compensating for) differences in this type by measuring the degree of randomness of the explanatory variables/factors involved (variables/factors that argue the magnitude of the calculated effect size) [48]. This kind of meta-analysis offers statistical models of the multivariate mixed random-effects type that estimate both the within-study and between-study heterogeneity (intrinsic and extrinsic variability of the reported clinical studies) [49].

In the realm of current opinions about the clinical value and opportunity of applying HA viscosupplementation compared to modern OA treatment options, the present paper intends to (i) offer a general meta-analytic view of the status of HA clinical use according to the recently published studies, but also to (ii) deeper evaluate the relation between the outcomes of HA-based treatments and the achievements reported for the treatment option that seems to install itself as the new referential in OA pain alleviation, the PRP intra-articular injections. In this regard, our approach consists of performing a typical meta-analytic study combined with meta-regression to generate and narratively exploit statistical models of the dependence of calculated effect sizes of clinical trials comparing HA viscosupplementation with recent (and for now “exotic”) alternative treatments. In this context, our work contributes to the formulation of a reasoned opinion regarding the opportunity of maintaining viscosupplementation among the clinically applicable treatment schemes, nuanced according to patient-related considerations, mainly the way he/she feels and subjectively reports joint pain. From a broader perspective, we aim to bring meta-analytic arguments regarding the advisability of continuing studies on improving viscosupplementation through better HA-based product formulation.

## 2. Results and Discussion

The present paper applies some methods of the meta-analytic investigation of literature data to assess the position of clinicians regarding the effects of use and the utility of continuing to prescribe and perform viscosupplementation of synovial liquid with HA in OA conditions. An intentionally heterogeneous set of papers published in the last ten years was collected and processed according to the PRISMA procedure [50]. The discrepancy in the set of papers originates from the wide variety of therapeutic attitudes toward OA pain alleviation methods, deliberately chosen to describe the present “landscape” of OA treatment trials. To gain value for practicing clinicians, our study then focuses on quantitatively comparing the effects offered by the two most commonly applied non-pharmacological treatments, viscosupplementation and intra-articular autologous PRP injections.

### 2.1. The “Wide Landscape” of Non-Surgical Osteoarthritis Treatments in Meta-Analytical Terms

**Figure 2:**
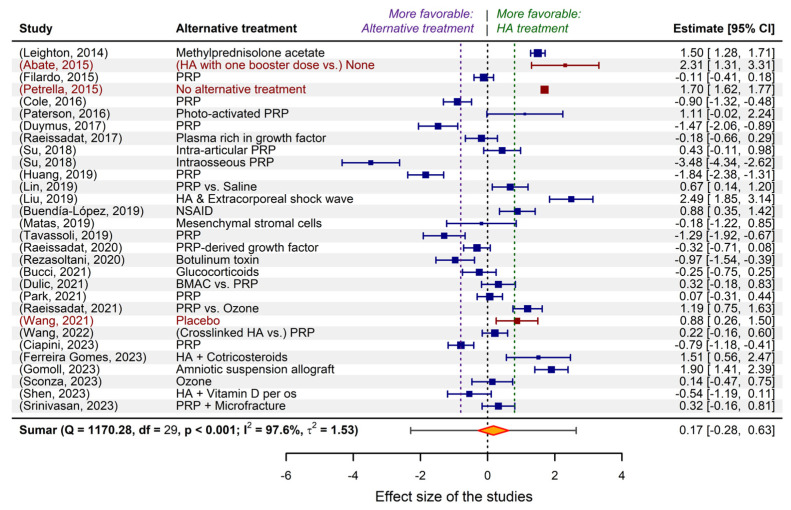
The “wide” meta-analytic comparison of the studies regarding OA pain alleviation, published in the 2014–2023 decade. Studies are inserted in chronological ascending order.

A meta-analysis statistically combines the results of different studies, aiming to benefit systematic reviews by generating summaries, both numerically and graphically. Several scenarios can be approached through meta-analysis, but all have in common the objective comparison, in abstract units, of the results reported in various sources of information (usually in scientific articles or in clinical trials). The comparison of the effects of distinct treatments can be made, for example, by comparing the effect sizes calculated as standardized mean differences (SMDs) of the measured results of the individual treatments. By doing so, a graphic way of comparing the reported effects of the treatments brought together in the systematic study of the literature carried out by us was obtained and depicted in Figure 2.

Three of the included studies do not express a genuine comparison against viscosupplementation by HA injections. One of them (Petrella, 2015) only reports the effect of applying or not applying the viscosupplementation. Another one (Abate 2015) discusses the effect of using booster doses of HA to perform viscosupplementation versus no other treatment. The third one (Wang, 2021) compares HA treatment with placebo intra-articular injections. These three studies (see the bibliographic details in Table S1, from the Supplementary Materials) were included in the meta-analysis to serve as a reference for the effect size, direction (sign), magnitude, and dispersity. Such a reference is necessary because the relative size of the treatment effect is measured using subjective assessment scales that are, in some cases, different from each other. In addition, statistical–mathematical modeling algorithms that use Bayesian inference methods require such starting information (including prior probabilities and their confidence intervals). The effect size of the viscosupplementation alone was chosen as the primary reference for the direction of the favorability of the treatment effects, while the dispersion reported for the booster treatment was used to calculate the potential magnitude of the viscosupplementation. A comparison with placebo injections was used to establish the sensitivity of the subjective assessment made by patients suffering from OA by means of joint pain assessment scales.

Obviously, the “wide” comparison reflected in Figure 2 has only a qualitative meaning, indicating the relative position of the alternative treatments on the dimensionless scale of standardized mean differences (effect size). The overall effect size (the orange rhombus) depicted in the bottom line of the forest plot is slightly positive (placed at +0.17), but it does not have a definite informational value for two reasons: (i) the heterogeneity of the alternative treatments that were subjected to comparison, and (ii) the large width of the prediction interval (represented by the color segment gray), which extends between −2.25 and +2.75, an interval in which the predicted cumulative effect could be placed anywhere. From a statistical point of view, such a large prediction interval expresses a poor precision of the comparison between treatments, while in clinical terms, it means a very low decidability on the opportunity to opt to elect a certain alternative treatment.

The two dashed lines delimit the range [−0.8, +0.8] on the abstract scale of the effect size beyond which the differences between the outcomes of the reference treatment (viscosupplementation with HA) and the alternative treatments are highly statistically significant.

The high level of randomness of the comparison results [51] is also expressed by the value of the heterogeneity statistic (I^2^ = 97.6%), which represents the percentage of variability between the included studies (the amount of dispersion caused by the pooled/combined interpretation of the studies). Numerical values of I^2^ exceeding 75% indicate substantial heterogeneity in the meta-analysis study. Its complement of up to 100% (=2.4%) is assimilated to the general variability caused by sampling error within the studies. The second measure of heterogeneity, the τ^2^ value (=1.53), describes the distribution of the true overall effect size about its central value (of 0.17) and can be considered an indicator of the neat randomness reflected by the meta-analysis. Its square root is an estimate of the standard deviation of the statistical distribution (presumed to be gaussian) of the true overall effect size.

A large heterogeneity in comparing studies usually results in misinterpretations of data and fitted models. Therefore, accurate and cautious validation must be conducted at all stages of the analysis. A biunivocal relationship exists between heterogeneity and study/model bias [52]. Generally speaking, bias consists of the difference between the observed, expected, or predicted value of an estimator and its true value. In meta-analysis, bias must be anticipated, if not prevented, to avoid poor validity of the results. Conflicting or, on the contrary, a disbalanced selection of studies to be analyzed represents one of the main sources of bias. There are three moments when bias can intervene in meta-analysis [53]: (i) the primary collection of studies; (ii) the selection of relevant studies; and (iii) the correct extraction of information from the selected studies. If, for the first and last of the three situations, there are algorithms to ensure their correct execution, the selection of the studies that will be analyzed represents the critical moment for the collaboration between clinicians and analysts when the selection criteria must be agreed upon.

Meta-analysis studies affected by large heterogeneity are difficult (and sometimes futile) to interpret. Various methods are available to reduce heterogeneity, such as (i) (re)grouping/clustering the included studies based on common characteristics; (ii) removing outlier studies; (iii) reconsidering the reference treatments against which alternative treatments are evaluated; (iv) limiting the number of included studies and then modeling the resulting selection. In this paper, the last-mentioned method will be used. To do this, study selection criteria must be defined.

### 2.2. Criteria Derived from a Clinical Perspective for Limiting the Selected Studies

The “wide” meta-analysis described above comprised 30 studies reflecting comparisons of 12 different alternative treatments, covering the three general strategies of combating OA pain (short-term pain relief, long-term suffering relief, and periodic control of inflammation through immunomodulation, respectively), and both the pharmacological and non-pharmacological types of treatment. Viscosupplementation using HA is involved in all studies as a reference treatment or as the only treatment.

Leaving aside the “exotic” alternatives of scientific interest rather than immediate application value, the idea that intra-articular PRP injections outperform HA-based treatments has been validated in clinical practice [54] and has been argued by recently published meta-analyses [55,56,57,58]. Due to the fact that the mechanisms by which these two treatment options lead to the relief of joint pain in OA are distinctly different [36], the selection criteria of the studies that have in common the specific comparison of HA and PRP treatments must consider the maximum possible number of details of clinical studies. The reason lies in the need to redo all the comparisons in the specific terms of meta-analysis and meta-regression. Therefore, the effect size of individual treatments must be (re)calculated, taking into account, in addition, information related at least to (i) patient-related data, (ii) the reported pain scores before and after treatments, and (iii) the administered doses and administration conditions (e.g., doses of HA and PRP should be similar in volume). In such a context, review articles should be excluded, as they do not include all the necessary details. Furthermore, few published articles contain all this information.

Based on the above-discussed selection criteria, out of the set of 30 published studies previously analyzed in Corpore, only eight lend themselves to meta-regression. One of them (Hegab, 2023) can be considered an outlier (because it refers to a very peculiar joint, the temporomandibular joint, which is injected according to a particular protocol), thus being eliminated as well. Consequently, the final selected set of studies, specifically dedicated to the HA and PRP pair of treatments, includes those listed in Table 1, along with the narrative results stated by the authors of the paper. The set of studies covers the entire typology of clinical situations in which the comparison of HA versus PRP is relevant.

### 2.3. Fitting Meta-Analytic Models to the Data Extracted from the Limited Selection of Studies

The relationship between treatment effects and the common characteristics of a set of clinical studies can be modeled in a way that is exploitable for further analysis and prediction. Meta-analytic models express the statistical–mathematical relationship between (i) the effect size (or outcomes) calculated based on the information extracted from the selected studies, (ii) a set of variables that are common to all the studies involved, and (iii) terms that describe the heterogeneity affecting the analysis in progress. In other words, the cumulative effect size, playing the role of the dependent variable (because it is calculated by the model), is related by a mathematical function to some predictors (also named explanatory variables) that act as independent variables (because they are directly or indirectly selected or manipulated in the conducted studies). The mentioned function is always parasitized by summative errors, which represent the heterogeneity of the studies subjected to the meta-analysis—errors that are, in turn, statistical functions. Classical meta-analytic models are usually fitted by using meta-analysis algorithms. They are strongly affected by the presence of bias in studies, whatever its source. If a significant value of heterogeneity is noticed for these models, its origin can be identified and evaluated by meta-regression, which is an extension of meta-analysis. Meta-regression can replace sub-group meta-analysis when the dependent variable is continuous [59] (pp. 267–271).

Pragmatically, meta-analytic modeling is carried out in the following steps: (i) data extraction from the selected studies or publications, finalized by a detailed and complete set of necessary data relevant for modeling; (ii) inventorying of available common information in order to identify the most relevant variables that can act as predictors; (iii) defining the role and the hierarchy (based on the weight of the role) of identified predictors into the model structure; (iv) postulating and actually generating the model by using the appropriate algorithms; (v) testing the adequacy of the model and assessing the statistical significance of its coefficients. In the following, we will detail this modeling process for the case of the meta-analytic study applied to the previously selected set of seven studies.

A number of 35 studies were selected from 32 articles taken from the set of 140, which were analyzed according to the PRISMA guidelines for the literature review. The entire set of articles is included in Table S1 of the Supplementary Materials. The information extracted from the selected studies is included in the Raw.data.txt ASCII file as an object that can be loaded into the R interpreter [60] by using the command:


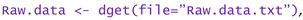


in the spirit of reproducible research [61]. After the first outlier articles were removed, 30 studies were retained for further meta-analysis. Based on this adjusted first selection, the “wide” meta-analytic comparison of the studies regarding OA pain alleviation was performed (depicted in Figure 2). The modeling was then applied to the narrow set of studies mentioned in Table 1. The ASCII file Data.selected.txt includes the corresponding R object, which can be loaded by using the R command:


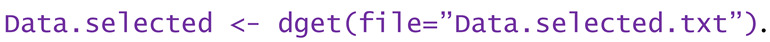


Different models were successively generated and diagnosed in order to obtain increasingly better accuracies of fitting the contribution of individual studies to the cumulative effect size in the context of reducing the model heterogeneity by selecting appropriate sets of predictor variables. With the same aim of reducing heterogeneity, different variables were included in the successive models to provide information regarding their randomness (the characteristics of their statistical distributions). These latter-mentioned variables were chosen to have clinical relevance, both individually and in pairs. The pairing of random variables took into account their nesting [62] according to clinical logic (see Section 4.4 of this paper).

Multilevel mixed-effects models with multiple random effects were fitted using the metafor R package. They were diagnosed, and, as a particular way of diagnosis, their prediction was represented graphically.

In the following, one of the models that offered good fitting precision and a reasonably low level of heterogeneity will be described. The model in question considered three moderator (predictor) variables: the pain score at the start, the patient’s average age, and the unit dose of HA or PRP administered as a single injection per treatment session. Three of the identified common variables in all studies and for all treated patients: unit dose, number of repeatedly administered doses, and the pain score at start, respectively, were taken into consideration to explain the random part and content of the model. The R command that generated the model had a structure similar to the simplified one below:


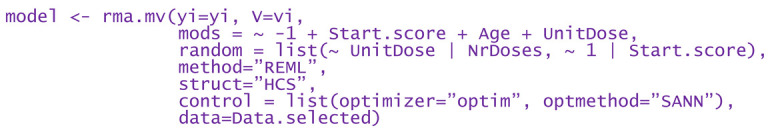


To facilitate reproducible research approaches, the R object corresponding to the above-exemplified model is included in the ASCII file Example.model.txt, which can be loaded into R by using the instruction:


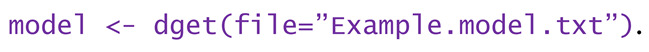


Th R script that exploits the model is included in the Supplementary Materials (the file Meta-analysis _ Exploitation of the model (Example).R).

**Figure 3:**
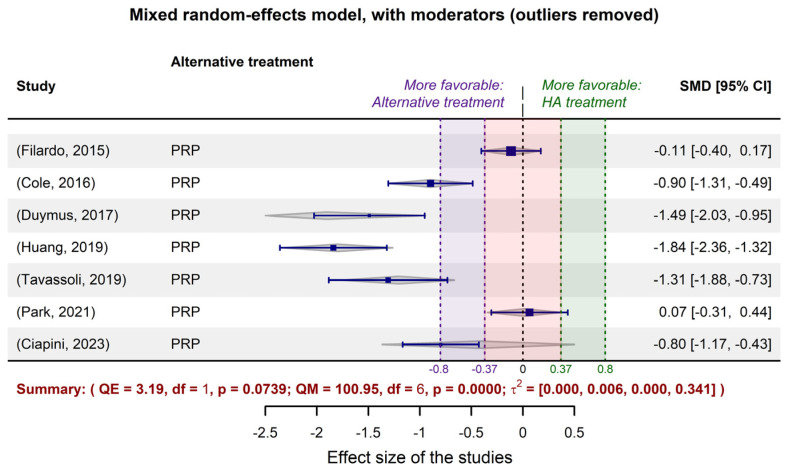
The result of meta-regression applied to the exemplified model that was fitted to the selected studies dedicated to PRP vs. HA efficacy meta-analysis. The gray rhombuses (“diamonds”) represent the confidence intervals corresponding to the individual studies, as predicted by the fitted model. Boundaries of clinical and statistical significance are drawn (the minimal clinically important difference (MCID = −0.37) and 0.8, the limit of high significant effect size) for both areas of favorability of the two types of treatment.

Figure 3 depicts the result (in the form of a “forest plot”) of the meta-regression applied to the above-discussed exemplifying model, performed considering two continuous moderator variables (WOMAC pain score before treatments and patient average age) and a discrete factor (administered unit dose). The graphic representation has a diagnostic role because it highlights the relationship between the confidence intervals calculated for the effect size of the individual studies (blue segments) and the confidence intervals of the effect size fitted by the model (gray rhombuses). The misalignments of the two confidence intervals are proportional to the fitting errors and express the effect of the heterogeneity of the studies on the predictive capacity of the model. In the particular case of the exemplifying model, the heterogeneity was reduced stepwise by taking into account the statistical parameters of the random variables mentioned in the model (pain score before treatments, unit doses, and number of administered doses; the last two are of discrete type). Residual heterogeneity is partially caused by the fraction of clinical studies inherently affected by uncertainty, which is expressed by the randomness of predictor variables (both moderators and those dedicated to the error parametrization).

In general terms, multilevel mixed-effects models derive from random-effects models defined by relations of the following type [63]:

(1) $${\hat{\theta }}_{j}=\beta_{i}M_{ij}+u_{j}+\epsilon_{j},\;\mathrm{weighted}\;\mathrm{by}\;w_{j}^{*}=\frac{1}{{\hat{\sigma }}_{j}^{2}+{\hat{\tau }}^{2}},\;\mathrm{where}\;u_{j}\sim N\left(0,\;\tau^{2}\right),\;\mathrm{and}\;\epsilon_{j}\sim N\left(0,\;{\hat{\sigma }}_{j}^{2}\right).$$

In the above equations, ${\hat{\theta }}_{j}$ is the calculated/estimated outcome (here the cumulative size effect) associated with the model to study *j*, $\beta_{i}$ are the coefficients of the fitted linear model related to the *i*-th moderator variable, $M_{ij}$ are the values of the *i*-th moderator variable in the *j*-th analyzed study, $u_{j}$ is the random “content” of the study *j*, $\epsilon_{j}$ is the sampling error associated with the *j*-th study, ${\hat{\sigma }}_{j}^{2}$ is the estimated variance for the *j*-th study, ${\hat{\tau }}^{2}$ represents the estimated variance of between-study heterogeneity that is unexplained by the considered moderators, and $\tau^{2}$ is the calculated between-study variability (the variance of the true effects size). In addition, multilevel models also take into account the nesting of the declared random variables (~ inner | outer correlated random effects) by means of correlation coefficients between the inner and outer ones. For one and two such pairs, the coefficients are called ρ and φ, respectively. Specifically, for example, ρ expresses the correlation between random effects of model variables that have the same level of the outer (if ρ<0) or of the inner (if ρ>0) imbriqued variable.

In particular, for the above-mentioned exemplifying model, its structure is as follows (y_j_ represents the calculated effect sizes of the j-th study, and v_j_ is their variance):


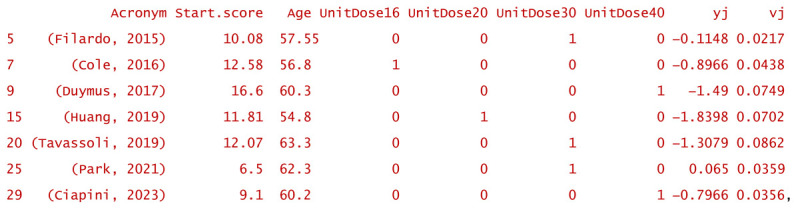


and the estimated coefficients, $\beta_{i}$, have the values:


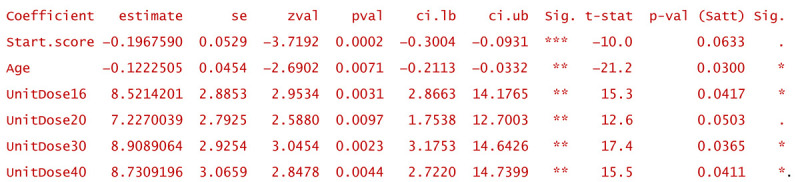


All coefficients are statistically significant (*p* < 0.05) and remain significant (*p* < 0.05) or near significant (*p* < 0.1) after applying the Satterthwaite correction for small samples. The coefficients expressing the heterogeneity, ${\hat{\tau }}^{2}$, are associated with the levels of a discrete inner factor (unit dose) and have the values ${\left.{\hat{\tau }}^{2}\right|}_{UnitDose16}=3\times 10^{-6}$, ${\left.{\hat{\tau }}^{2}\right|}_{UnitDose20}=5.9\times 10^{-3}$, ${\left.{\hat{\tau }}^{2}\right|}_{UnitDose30}=6.3\times 10^{-8}$, and ${\left.{\hat{\tau }}^{2}\right|}_{UnitDose40}=3.4\times 10^{-1}\;$(see also the summarizing line in Figure 3). Except for the last value listed, which is that for unit doses of 40 mg per injection, the injected dose does not contribute significantly to explaining the heterogeneity of the meta-analytic study. The coefficient of the correlation between the inner and outer random variables, ρ, is negative and has the value of −0.99846 (being basically −1), which, because both the inner and outer variables are discrete, indicates that multiple doses are favorable at any unit dose. In other words, for modeling, it is not necessary to consider the nesting of the two variables.

In conclusion, without significantly losing the accuracy of the modeling and without omitting the identification of important sources of heterogeneity, the model can be simplified. For example, the most frequently used unit dose (30 mg per injection) can be chosen, and its interdependence with the number of doses can be abandoned. Such numerical experiments (or “virtual studies”) can be performed using the predict.rma function of the metafor package, applied to either the original model or the simplified model. In the following, some examples of numerical experiments are provided.

− Consider only unit doses of 30 mg HA or PRP per injection and no nesting:

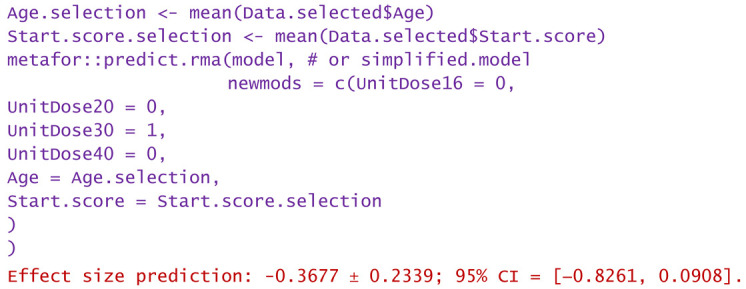

− Same as above, but only for patients over 60 years:

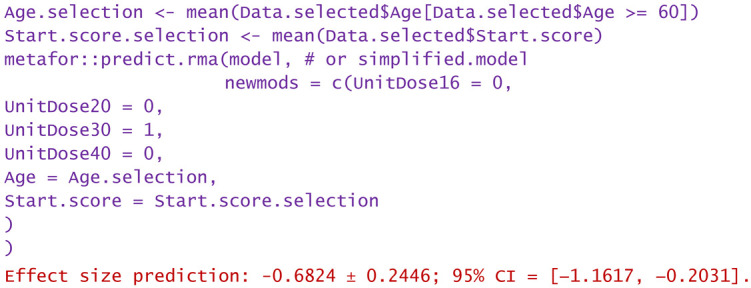

− Same as above, but only WOMAC pain scores at the start between 5 and 10:

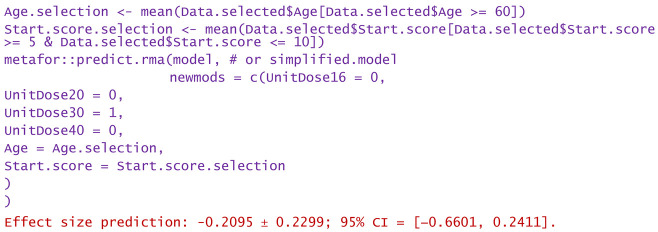

− Same as above, but only the highest start WOMAC pain scores in the range of 5 to 10:

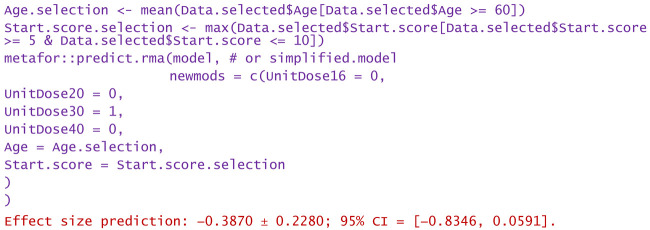

− For a patient aged 80 and reported WOMAC pain score of 12:

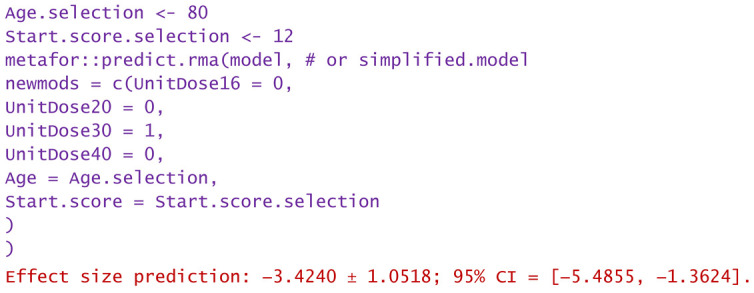

− A dose of 16 mg was administered to a patient aged 50 and reported WOMAC of 6:

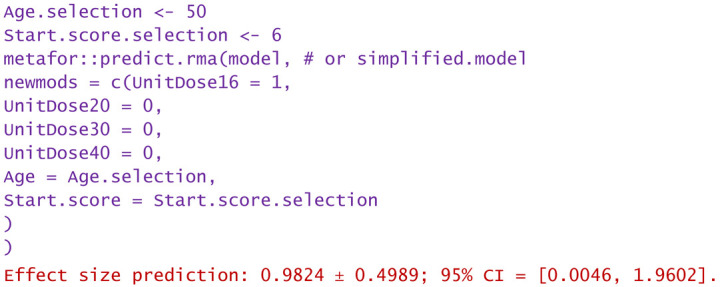


Predictions are reported as the calculated value of the effect size in the hypotheses mentioned in the statement. Using them, clinicians can decide the degree of favorability of applying a certain injectable treatment in a wide variety of scenarios. If negative values are predicted, the alternative treatment (in particular, the one using PRP) is more favorable in that circumstance. Positively predicted effects size indicates viscosupplementation as being more favorable. The higher the predicted absolute numerical values are, the more favorable the choice of the treatment in question.

The more complex the validated meta-regression models are, the more sophisticated queries they can support and the more elaborate and useful guidance they can provide. Therefore, detailed meta-analyses are useful, but only if accurate statistical validation of them is performed. However, the richer the meta-analysis/meta-regression is in predictor variables, and the more such variables are included in the models, the more carefully and deeply their statistical validation needs to be conducted. Also, detailed checks must be performed in the preliminary stages of the meta-analysis. One such check is the publication bias of the articles/clinical studies considered and selected for modeling. The next section will address this issue, both graphically and using the Egger test.

### 2.4. Publication Bias

Prejudgments and self-censorship are not uncommon in the process of publishing scientific papers. According to one of them, only (or predominantly) those studies reporting results with high statistical significance are published, disregarding the fact that (i) apparently less statistically convincing studies contain useful information, (ii) sometimes inadequate statistical investigations are carried out or are poorly interpreted, and (iii) even the studies that do not confirm the initial (statistical) hypotheses contain valid arguments. As a consequence, especially the optimistic or partisan articles that are available to the scientific community or as official/formal clinical trials, the other studies remain in the area of gray literature. This fact is detrimental to meta-analysis, as it determines the bias of the publications. For example, predominantly partisan studies related to new clinical attitudes can be found in the last decade, inducing the idea that older ones are inadequate, outdated, or comparatively even dangerous. The meta-analysis will, in such cases, be artificially/incorrectly unbalanced and will suggest an exaggerated heterogeneity, the sources of which are not found in the investigated data. For this reason, especially when the meta-analysis indicates large heterogeneities, it is necessary to check the degree of bias of the data that were collected from the available publications.

A widely used method to assess the effect of publication bias on meta-analysis results is the trim-and-fill procedure [64]. Due to its ability to explain the extent of heterogeneity in a collection of studies, it can also be used to discriminate between different models generated by meta-regression. The trimt-and-fill procedure uses the funnel plot (a scatterplot that relates the precision of individual studies to their outcome) to detect publication bias and suggest ways to compensate for it.

**Figure 4:**
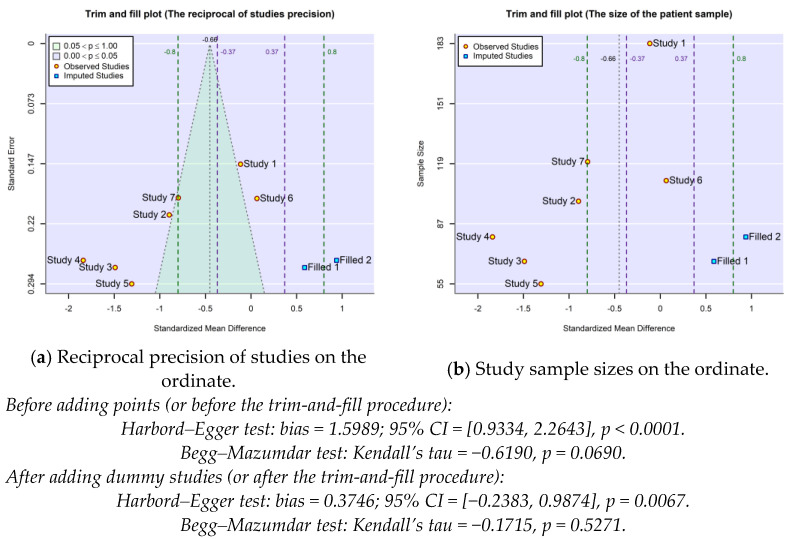
The funnel plots associated with the equal-effects (method=”EE” in rma.uni function of metafor package) meta-regression performed for the exemplifying model discussed in the previous sections of the paper after applying the trim-and-fill procedure. The precision (**a**) and robustness (**b**) of studies are depicted as a function of their calculated effect size. Harbord–Egger and Begg–Mazumdar asymmetry tests are included for both pre- and post-adjustment.

Figure 4 presents two variants of the funnel plot: one dedicated to the study precision (the inverse of the study precision as a function of their calculated effect size) and the second dedicated to the study robustness (the sample size as a function of the calculated effect size). Both are drawn after completing the set of studies through the trim-and-fill procedure by adding two dummy studies. The sparse and wide distribution of study points indicates a large heterogeneity of the included studies. Practically all of them are placed outside of the “cone” of the 95% confidence interval, suggesting that the between-studies variability is excessive, and perhaps a wide selection should be conducted. A probable cause of the heterogeneity derives from the large difference between the number of patients included in the selected studies (a three-fold difference). Due to the robustness disbalance of studies (the quasi-neutral study 1, (Filardo, 2015), being the more robust), the cumulative effect size (of −0.656 abstract units) still has a modest statistical relevance, even if it exceeds the threshold of MCID. The imbalance is also reflected in the figures of asymmetry tests, as the presumed bias remains positive even if a larger number of studies are placed to the left of the equivalent study represented by the calculated cumulative effect size. After the trim-and-fill procedure, the extended set of studies (the original ones plus two dummy studies appropriately chosen by the algorithm) seems to exhibit a four-fold reduced bias, from about 1.6 to about 0.4 (see the values of the Harbord–Egger test in Figure 4).

**Figure 5:**
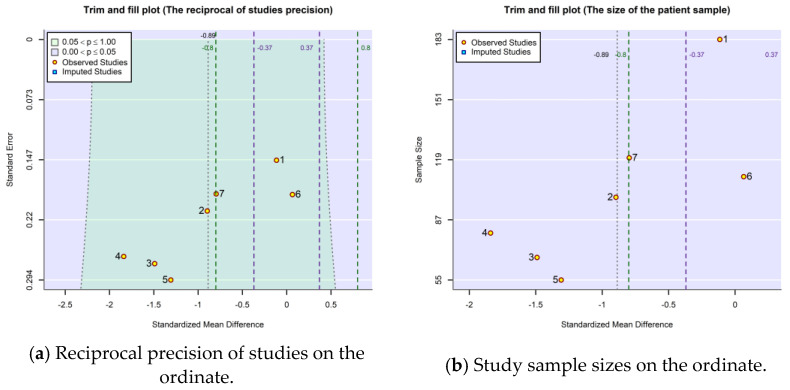
The funnel plots associated with the random-effects model (method=”REML” in rma.uni function of metafor package) when the pseudo confidence interval also considers the amount of residual heterogeneity estimated by the model ($\pm 1.96\sqrt{{SE}^{2}+{\hat{\tau }}^{2}}$).

In the context of the small volume of available studies and because the dispersion of their results remains large even after the trimming and filling procedure, the heterogeneity of the studies most likely stems not only from publication bias but also (and probably predominantly) from the variability of the studies selected to compare the effects of HA and PRP treatments. To confirm that the wide dispersion of study points in the funnel plot is mainly caused by between-study heterogeneity and only to a small extent by publication bias, in Figure 5, the pseudo-confidence interval (which delimits the central green area) was incremented by the value of estimated residual heterogeneity. Compared to Figure 4 (generated using the R object associated with the equal-effects model included in the Exmple.model.fix.EE.txt file in Supplementary Materials), the trim-and-fill procedure did not find it necessary to add, this time, additional study points. The meta-analytic random-effects model (whose R object is found in the Exmple.model.fix.REML.txt file), which is capable of estimating the residual heterogeneity, ${\hat{\tau }}^{2}$, was fitted using the restricted maximum-likelihood estimator (REML) applicable for linear mixed models. The calculated cumulative effect size became more favorable for PRP treatment, with the jump between the two models being from −0.66 to −0.89 simply by accounting for the identified/predicted residual heterogeneity.

In conclusion, based on the set of selected files, the ability to decide on the favorability of PRP treatment versus viscosupplementation remains modest. The reason is that the studies from the last decade that report all the details necessary to conduct an independent meta-analysis are both few and discordant.

### 2.5. On the Usefulness of Meta-Analytical Modeling of Viscosupplementation and Its Alternatives

Medical bioinformatics has emerged as a toolkit of clinical utility. In the taxonomy of its tools, meta-analysis/meta-regression represents a statistical way of systematizing comparative information extracted from clinical studies (mainly of retrospective type) and clinical trials (that were particularly designed ab initio).

Meta-analysis is able to highlight and explain the effects of compared treatments, while meta-regression provides relevant quantitative indices calculated for peculiar clinical contexts based on postulated and then statistically validated dependencies. In order for the modeling results to be truly useful, the premises must be established by clinicians, and the algorithms must be used judiciously and creatively by statisticians. Our work represents an illustration of the pairing of such skills.

The meta-analytical/meta-regression study we have performed started with the hypothesis that viscosupplementation should remain a piece in the clinical armamentarium of orthopedists, in spite of the partisan records that have been recently published. The rationale behind our hypothesis is based on the assumption that the new (K)OA treatment options, some of them “exotic,” exceed in complexity the intra-articular administration of a pharmacologically certified product that is biochemically similar to synovial fluid and consequently raises insignificant cyto- and bio-compatibility issues. Therefore, our reasons are of a practical nature, and we do not contradict the scientific foundations of the new treatment alternatives for the pain caused by (K)OA.

At the current stage of mainstream clinical practice, there is only one already established challenger of viscosupplementation, the autologous PRP. For this reason, after sketching the landscape of published alternatives (by means of a “wide” meta-analysis based on a selection that is amalgamated per se), we focused on the comparison between the administration of exogenous HA and autologous PRP, respectively. In this regard, we used a narrow selection of published studies (seven out of 30) devoted to the alleviation of KOA pain. In this selection, we have tested postulated meta-regression models with individual and nested predictor variables of clinical relevance, making the most of the continuous and discrete numerical data found in the seven selected articles. We have further outlined various clinical scenarios, which are not very complicated but relevant in daily practice, on which we conducted meta-regression in order to numerically correlate patient-related peculiarities with treatment details. The usefulness of this approach derives from the possibility offered to clinicians to decide both the nature of the treatment applied (intra-articular injections with either HA or PRP) and the administration conditions (dosage, number of doses, recurrence) in a statistically substantiated direct connection with a specific patient.

In general terms, our clinically argued meta-analytic/meta-regression study exemplifies how literature data can be capitalized to extract and exploit relevant information for practitioners. In particular, we have argued that the effectiveness of treatment based on HA differs only circumstantially from that provided by PRP. For this reason, taking into account the statistically revealed undecidability in the particular case of comparing HA versus modern alternatives (excepting, perhaps, autologous PRP) of (K)OA treatment, we consider that sterile HA in the form of a gel or diluted colloidal solution should retain its status as an efficacious injectable treatment.

Naturally, our meta-analytic/meta-regression approach aimed at particular situations and tried to address the difficulty raised by the paucity of data available in the scientific literature, data that would have allowed for the recalculation of all statistical details required for an argumentative study (such as ours, which compared the efficacy of viscosupplementation products based on exogenous HA with the efficacy of autologous PRP). However, over the last decade, the quality and completeness of clinical data reported in published articles have significantly improved. In this context, the data processing template that we tested allows for much more nuanced future approaches. In schematic terms, starting from a global meta-analysis of the targeted clinical topic (or of any other one), the study can be progressively customized until reaching a data set that allows a fair choice of predictor variables. Based on them, a meta-regression study can be launched, the complexity and relevance of which depend only on the number of accessible variables and the inventiveness of the clinician–analyst pairs. This will result in libraries of models that will play the role of tools for the clinician.

Even though it was not designed to make significant strides toward medical informatics, our work suggests a useful way to process clinical data. Meta-analysis and meta-regression are part of the statistical–mathematical techniques through which artificial intelligence is trained to offer predictive solutions in the spirit of machine learning. Therefore, our modeling approach can represent a prelude to a more customized and complex approach to clinical data processing, “in tune with the times.” To facilitate interdisciplinarity, we approached the reproducible research policy. In this way, the procedure we followed in our work can be widely used by researchers interested in topics related to medical treatment comparison and comparison modeling.

### 2.6. Notes in the Spirit of Self-Criticism

The study we conducted was limited by the lack of comprehensive information provided by the original papers available in scientific repositories, some of which were biased regarding the effectiveness of viscosupplementation. We did not use data taken from official clinical trials or systematic reviews, such as those from the Cochrane library. This is why the number of variables considered in the meta-analysis/meta-regression was inherently small. We tried to compensate for this fact by wisely choosing the predictor variables and checking if the nesting of some of them is statistically relevant (although the nesting seems to be clinically relevant).

In our work, we used public-domain programming software and algorithm libraries. We consider them more versatile compared to “menu-driven” software applications, even if they are more difficult to use due to the need to program the applications ourselves in a computer language. Their versatility derives from the access they allow to all the details of the implemented algorithms, and, in this respect, the R language and R packages are recognized and appreciated in the world of both scientists and statisticians.

## 3. Conclusions

Even though newer alternatives for the treatment of (K)OA seem to offer better results (reported as effect size in contrasting clinical studies) compared to viscosupplementation, HA-based treatments retain some of their advantages, mainly stemming from the similarity of exogenous HA to the synovial fluid that is physiologically present in the joints. Recent articles call into question the appropriateness of HA-based treatments of (K)OA (for example, reference [65], which states: “The findings do not support the broad use of viscosupplementation for the treatment of knee osteoarthritis.”). This paper tried (i) to put viscosupplementation in a wider context of the treatments proposed or argued in the last ten years and then (ii) to compare viscosupplementation with its main clinically feasible alternative, the administration of autologous PRP. The overall aim was to help build an opinion on whether HA-based products are worth manufacturing, developing/formulating further, or whether they have come to an end. Our conclusion, which is not surprising and in line with other opinions, is that the clinical studies published in the scientific literature do not yet decisively lean towards the abandonment of viscosupplementation but that autologous PRP is a more feasible alternative for severe cases and for elderly patients. As a tool for clinicians, we have developed exemplifying meta-analytic models, which can be useful in substantiating a treatment option. The models in question can be extended if additional clinical data are accumulated, either through the current interaction with the patients (observational or case-control studies) or through comparative clinical studies (treatment trials).

## 4. Methods of Statistical Investigation

Our work focused mainly on the literature that reports systematic studies on the (K)OA medical conditions treated comparatively by viscosupplementation with neat HA (in the form of gels or elasto-viscous colloidal solutions) and by its newer alternatives. Original and review articles published on this topic were collected for the last ten years (2014–2023) from four of the top-rated scientific paper repositories. The full list of the 140 consulted articles is included in Table S1 of the Supplementary Materials.

**Figure 6:**
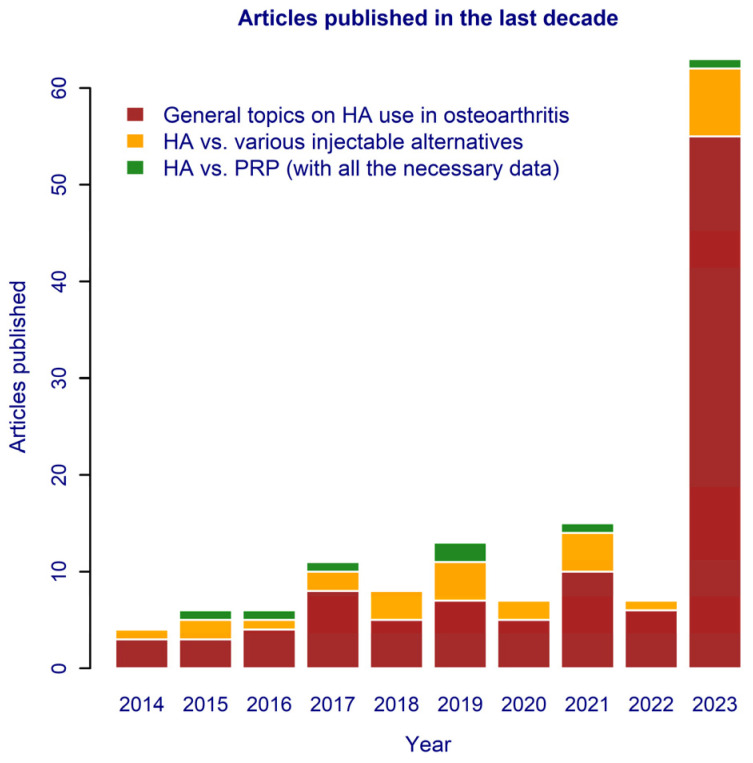
Dynamics of publications related to the use of HA in osteoarthritis and the topics covered during the 2014–2023 decade.

The publication dynamics during the considered decade are depicted in Figure 6, together with the coverage of the topic regarding HA applications in OA and in joint disease-related subjects. Except for the year 2023, the fraction of the number of articles dedicated to the comparison of injectable forms of HA against its challengers remained steady. Unfortunately, articles that provide all the necessary details for meta-analysis/meta-regression are few (seven in ten years) and mainly refer to the pair of HA and PRP.

### 4.1. The Retrospective Study in PRISMA Terms

The present study uses the PRISMA (Preferred Reporting Items for Systematic Reviews and Meta-Analyses) philosophy to construct a meta-analytic view of the position of HA use in the landscape of modern alternatives that could replace viscosupplementation in clinical practice. It combines retrospective literature study, meta-analysis, and meta-regression approaches, even if it is not a genuine systematic review but a gradual processing of clinical information in the PRISMA spirit.

**Figure 7:**
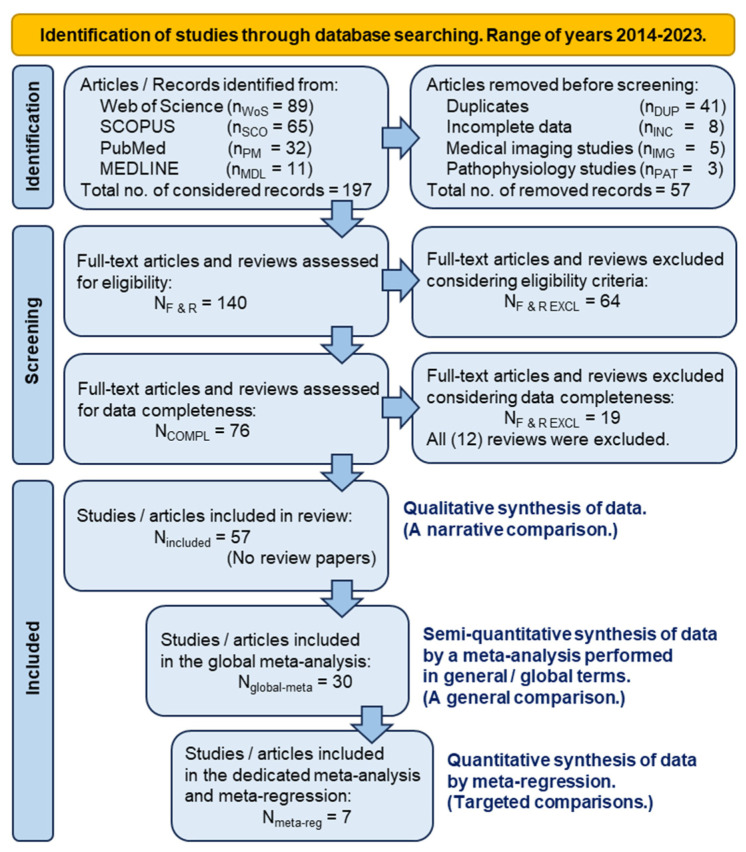
The PRISMA 2020 flow diagram of the collection, screening, and exploiting of information in the analyses carried out in the present work.

The phased selection and processing of the information found in the articles of potential interest for the successive stages of our study was performed according to the PRISMA guidelines in their 2020 statement version [50]. The PRISMA flowchart of the procedure of information selection is depicted and quantitatively detailed in Figure 7 (which was generated based on the template from the WEB address https://www.prisma-statement.org/prisma-2020-flow-diagram, accessed on 6 May 2024). Since the objective of our study was not to carry out a comprehensive systematic review, the Prisma 2020 Checklist was only selectively covered.

The initial grouping of articles was subjected to a systematic analysis in order to identify those that provided the full set of information and numerical data needed to successfully apply meta-analysis and meta-regression to the calculated effect size of the (K)OA comparative treatment. Following the two-stage systematic analysis, only about 25% of the initially selected articles were retained. As a general rule, review papers were excluded. A second group of articles was selected from the first one, considering the presence in their content of a reported comparison between the effects of viscosupplementation (as a reference treatment) and the classically prepared PRP (as an alternative treatment). This second criterion was chosen considering the immediate clinical applicability of the two treatment options. Information on the number of patients involved, the initial and final pain scores reported, and the standard deviation of the corresponding scores were considered mandatory for the second selection stage, which acted as a drastic constraint (only about 5% of the collected articles were qualified under these conditions for the final analysis and modeling).

Tasks related to the retrospective study were seconded by R packages *metagear v0.7* [66], *PRISMA2020 v1.1.1* [67], and *PRISMAstatement v1.1.1* [68], under the R language (*R version 4.4.0*) [60], and the integrated development environment (IDE) RStudio 2024.04.1+748 for Windows [69]. The R interpreter, RStudio IDE, and all packages are freeware software applications that can be downloaded from the WEB portals https://cran.r-project.org/, accessed on 6 May 2024 and https://posit.co/products/open-source/rstudio/, accessed on 6 May 2024. Running *R base’*s *sessionInfo* function on the computer we used provided the details of the hardware and workspace that were involved in processing the data, as follows (*quod infra scriptum in effectu est facimus indubiam, etiamsi est abbreviationem*):


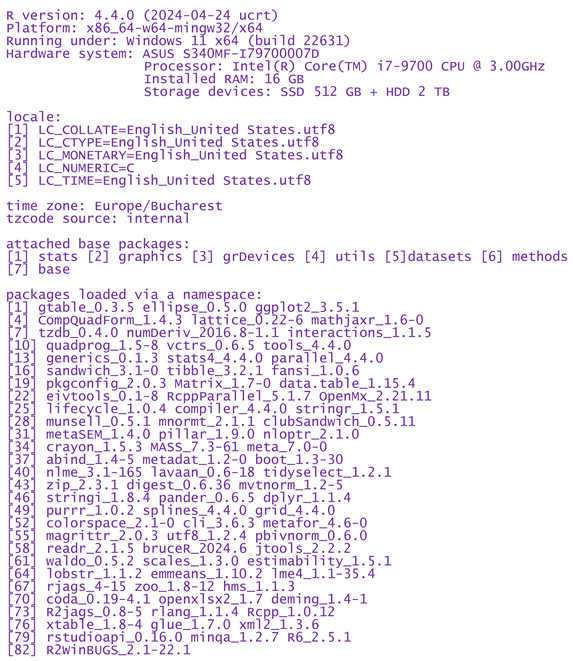


### 4.2. The Process of Selecting Articles of Interest

From the pool of 197 references collected, 140 were systematized as a first step. Next, two successive screening steps yielded a group of 57 original papers that included the information needed to compare HA-based treatments of (K)OA with different types of alternative treatments. The screening was conducted starting with the requirement of gathering all the numerical data needed to calculate the effect size of the comparison treatment. As an additional step, of the 57 articles, 30 studies were identified as discussing significant alternative treatments. Among them, seven are dedicated to the alternative pair, HA versus PRP.

### 4.3. The Semi-Quantitative Synthesis of Comparison Data by Meta-Analysis

A meta-analytic investigation was performed first on the heterogeneous set of 30 studies dealing with a diversity of alternatives to HA-based treatment (12 in number). All three types of (K)OA pain alleviation (by means of anti-inflammatory medication, intra-articular injections, and immunomodulators, respectively) were considered. In addition, three non-comparative/non-alternative reports on the effect of HA on pain were included, one against a placebo-type intra-articular injection and two considering no alternative treatment: HA alone and HA booster doses, respectively. Their role in the meta-analysis was to serve as a referential benchmark for the effect size and magnitude of the HA-based viscosupplementation.

Meta-analysis and adjacent calculations were performed using scripts written in the R language (*R version 4.4.0*) [60] under the RStudio IDE (RStudio 2024.04.1+748 for Windows) [69]. The following R packages were used: *metafor* v4.6-0 [70], *clubSandwich* v 0.5.10 [71], *TOSTER v0.8.3* [72], and their dependencies for optimization calculation and graphic representation, together with several complementary packages like *compute.es v0.2-5* [73], *effectsize v0.8.8* [74], *esc v0.5.1* [75], and *openxlsx2 v1.7* [76].

The effect size of all treatment options, comparative or not, was calculated under the hypothesis of unequal variance [77], as standardized mean difference (SMD), with the necessary correction for small samples [59] (pp. 157–158, 582–584), ω, computed in R by using the expression (deduced from the suggestions of the reference [78]):


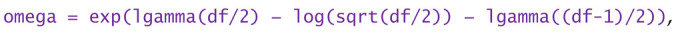


where the degree of freedom is df = (n_treatment_ − 1) + (n_reference_ − 1), taking into account the number of patients treated with HA and with the alternative option, respectively. Two types of thresholds were used to judge the magnitude of the significance of the calculated effect size, one considering statistical terms and the second considering clinical terms, both expressed in abstract units. Statistically, the threshold was 0.8, which, according to Cohen’s convention, means “significantly large effect size values” [79]. All effect sizes exceeding ±0.8 were considered large enough to provide evidence of a statistically significant difference between the treatment options being compared. In clinical terms, the threshold was adopted from reference [66] (Web appendices 6 and 5) at a value of −0.37. It represented the *minimal clinically important difference* (MCID) between the treatment options compared in cases of KOA when the reference treatment was viscosupplementation and the outcome was the post-treatment pain intensity. Calculated effect size values exceeding ±0.37 were considered to significantly tip the balance toward the alternative treatment (in the case of negative values) or towards the HA-based treatment (when the effect size values were positive). The two conventions mentioned regarding the thresholds were applied both for the effect size in individual studies and for the cumulative effect size calculated by meta-analysis. Testing whether the calculated effect sizes exceeded the thresholds of statistical and/or clinical interest was performed using the procedure of two one-sided tests (TOSTs), offered by the functions in the TOSTER R package [72], which also provide the confidence (and prediction) interval for the standardized mean difference between the compared treatments. Finally, the statistical decision was objectified based on the position of the threshold values in relation to the calculated confidence interval (by means of calculated *p*-values but also by graphical representation).

### 4.4. The Quantitative Synthesis of Comparison Data by Dedicated Meta-Analysis and Meta-Regression

In order to specifically compare the outcomes of the two most clinically applied injectable pain treatments in cases of (K)OA, (i) the HA-based viscosupplementation of synovial fluids, and (ii) the chondrocyte stimulation by PRP, information from the seven original papers devoted to this topic in the last ten years was used. The apparently small number of these articles is the consequence of a preliminary stage in which “outlier studies” were eliminated from the selection obtained in the screening stage of the PRISMA procedure. Those studies referring to discordant clinical situations, such as the cases of joints with different net volumes of synovial fluid (as temporomandibular joints compared to the knee) or unsimilar injection regimens (too different sequences, frequencies, and/or cadence of administration) between HA and PRP treatment, were considered “outliers.” In statistical terms, keeping “outliers” in the selection leads to an artificial increase in the variability of the results, while in clinical terms, there is obviously a modest plausibility to the comparison. Therefore, in both circumstances, inadequate statistical models will result.

Useful information for interpreting the direction and amplitude of differences between the compared treatments (such as HA versus PRP) can be obtained by fitting meta-analytic models. Such models mathematically relate the meta-analyzed outcome (here, the effect size of the considered studies) to a set of influencing factors selected from those that were included in the systematic PRISMA retrospective study. In our work, mixed-effects models were considered, which include both individual and hierarchical variables acting as predictors (also called moderators), some of which are affected by randomness. In simple terms, moderators are those study variables that are believed, based on clinical experience, to be able to explain the variability in the effect size of the individual studies being compared by meta-analysis (or the heterogeneity of the fitted model). Moderators can be continuous variables (such as pain scores, patient age, body mass index, gender percentage, etc.) or discrete factors (like the unit doses imposed by the characteristics of the administered product, the number of administered doses, the administration interval, etc.). Discrete factors are categorical variables, and some of them may be interrelated according to a logical hierarchy of interdependence. As an example, the number of administered doses depends on the amount (volume, mass, concentration) of the unit dose of the product chosen to be administered. In statistical terms, the two factors mentioned are imbricated (nested), one of them being the outer one (here the number of doses) and the other the inner one (here the unit dose, which is nested by the number of doses). In addition, the rule for selecting the levels of nested moderators (influencing factors) may operate as a function of, or correlated with, another variable or factor that is intrinsically random. In this context, which is frequently encountered in clinical practice, the grouping of nested factors must also be treated as a random variable. For this reason, the model fitting algorithm must be “informed” about the randomness of the group of nested factors as well as about the random variable that induced the respective status of the grouping.

Due to the complexity of the fitting situations, mixed-effects models are highly sensitive to the way of defining the type, nature, and possible intercorrelation of their variables. If these are poorly “explicit,” the model will hardly converge to a solution, will be modestly adequate, will provide coefficients with modest levels of significance, and will only vaguely adjust the size effect in the meta-analysis. In addition, optimization algorithms are used in the fitting procedure of mixed-effect models. In turn, these algorithms are sensitive to the starting conditions (the initial parameters of the “problem” description) and to the size of the variation step. In order to reduce the risk of poor fitting, we used optimization algorithms that possess self-adjustment mechanisms (Hooke–Jeeves derivative-free minimization algorithm and simulated annealing method). Some assumptions must be satisfied when mixed-effects models are fitted, like the linearity of the dependence of outcomes on influencing variables/factors, the absence of outliers in terms of variable value/factor levels, the absence of multicollinearity between variables or factors, and the normal distribution of the model’s residuals.

The *metafor* R package includes versatile fitting functions applicable to the generation of mixed-effects models in the context of quantitative meta-analysis. It also includes functions for diagnosis, graphic representation, and interpretation of this type of model. In our work, we used the functions *rma.uni*, *rma.mv*, *escalc*, *predict.rma*, *profile*, *forest*, *regplot*, and *funnel*. We also resorted to the *coef_test* and *conf_int* functions of the *clubSandwich* package. Statistical significance for all tests, analyses, and predictions was set at *p* < 0.05.

## Figures and Tables

**Table 1:** The studies were selected for statistical–mathematical modeling by means of meta-analysis and meta-regression techniques.

Chronological Order	Study	Narrative Outcomes of Compared Treatments from the Perspective of Their Inclusion in the Meta-Analysis/Meta-Regression Investigation
1.	(Filardo, 2015)	No superiority of PRP treatment over viscosupplementation.
2.	(Cole, 2016)	No substantial difference between PRP and HA treatments in terms of the pain score on the WOMAC pain subscale.
3.	(Duymus, 2017)	PRP is more successful than HA in the treatment of mild to moderate KOA.
4.	(Huang, 2019)	PRP performs better than HA in the early stages of KOA.
5.	(Tavassoli, 2019)	PRP is twice as effective in reducing pain as compared to HA, but after two injections at three-week intervals.
6.	(Park, 2021)	PRP acts significantly better in reducing KOA pain, even six months after the injection when the viscosupplementation effect decreases.
7.	(Ciapini, 2023)	The combination of PRP and HA outperforms treatment with HA alone in KOA.

## Data Availability

The data presented in this study are available on request from the corresponding author.

## Supplementary Materials

The following supporting information can be downloaded at: https://www.mdpi.com/article/10.3390/gels10070481/s1, Table S1: The full list of consulted articles; R objects and scripts in ASCII format (for reproducible research approaches): Raw.data.txt, Data.selected.txt, Example.model.txt, Exmple.model.fix.txt, Meta-analysis _ Exploitation of the model (Example).R.
