# Supplementary material for: A Meta-Analytical Way of Systematizing the Use of Hyaluronan Gels for the Relief of Osteoarthritis, Compared with New Treatment Alternatives
Source: Gels. 2024 Jul 20;10(7):481. doi: 10.3390/gels10070481 (PMC11276564; doi:10.3390/gels10070481)
Supplement: Supplementary file 1 [file gels-10-00481-s001.zip › Supplementary Materials _ Table S1.pdf]

Supplementary Materials

A meta-analytical way of systematizing the use of hyaluronan gels for the relief of osteoarthritis, compared with new treatment alternatives

Table S1. The full list of consulted articles (in ascending chronological order).

No.	Acronym	Reference details
1.	(Andia, 2014)	Andia, I.; Abate, M. Knee Osteoarthritis: Hyaluronic Acid, Platelet-Rich Plasma or Both in Association? *Expert Opin. Biol. Ther.* **2014**, *14*, 635–649.
2.	(Bannuru, 2014)	Bannuru, R.R.; Vaysbrot, E.E.; Sullivan, M.C.; McAlindon, T.E. Relative Efficacy of Hyaluronic Acid in Comparison with NSAIDs for Knee Osteoarthritis: A Systematic Review and Meta-Analysis. *Semin. Arthritis Rheum.* **2014**, *43*, 593–599.
3.	(Cross, 2014)	Cross, M.; Smith, E.; Hoy, D.; Nolte, S.; Ackerman, I.; Fransen, M.; Bridgett, L.; Williams, S.; Guillemin, F.; Hill, C.L.; et al. The Global Burden of Hip and Knee Osteoarthritis: Estimates from the Global Burden of Disease 2010 Study. *Ann. Rheum. Dis.* **2014**, *73*, 1323–1330.
4.	(Leighton, 2014)	Leighton, R.; Akermark, C.; Therrien, R.; Richardson, J.B.; Andersson, M.; Todman, M.G.; Arden, N.K.; DUROLANE Study Group NASHA Hyaluronic Acid vs. Methylprednisolone for Knee Osteoarthritis: A Prospective, Multi-Centre, Randomized, Non-Inferiority Trial. *Osteoarthritis Cartilage* **2014**, *22*, 17–25.
5.	(Abate, 2015)	Abate, M.; Vanni, D.; Pantalone, A.; Salini, V. Hyaluronic Acid in Knee Osteoarthritis: Preliminary Results Using a Four Months Administration Schedule. *Int. J. Rheum. Dis.* **2015**, *20*, 199–202.
6.	(Altman, 2015)-1	Altman, R.D.; Schemitsch, E.; Bedi, A. Assessment of Clinical Practice Guideline Methodology for the Treatment of Knee Osteoarthritis with Intra-Articular Hyaluronic Acid. *Semin. Arthritis Rheum.* **2015**, *45*, 132–139.

7.	(Altman, 2015)-2	Altman, R.D.; Manjoo, A.; Fierlinger, A.; Niazi, F.; Nicholls, M. The Mechanism of Action for Hyaluronic Acid Treatment in the Osteoarthritic Knee: A Systematic Review. *BMC Musculoskelet. Disord.* **2015**, *16*, 321.
8.	(Filardo, 2015)	Filardo, G.; Di Matteo, B.; Di Martino, A.; Merli, M.L.; Cenacchi, A.; Fornasari, P.; Marcacci, M.; Kon, E. Platelet-Rich Plasma Intra-Articular Knee Injections Show No Superiority Versus Viscosupplementation: A Randomized Controlled Trial. *Am. J. Sports Med.* **2015**, *43*, 1575–1582.
9.	(Henrotin, 2015)	Henrotin, Y.; Raman, R.; Richette, P.; Bard, H.; Jerosch, J.; Conrozier, T.; Chevalier, X.; Migliore, A. Consensus Statement on Viscosupplementation with Hyaluronic Acid for the Management of Osteoarthritis. *Semin. Arthritis Rheum.* **2015**, *45*, 140–149.
10.	(Petrella, 2015)	Petrella, R.J.; Wakeford, C. Pain Relief and Improved Physical Function in Knee Osteoarthritis Patients Receiving Ongoing Hylan G-F 20, a High-Molecular-Weight Hyaluronan, versus Other Treatment Options: Data from a Large Real-World Longitudinal Cohort in Canada. *Drug Des. Devel. Ther.* **2015**, *9*, 5633–5640.
11.	(Cole, 2016)	Cole, B.J.; Karas, V.; Hussey, K.; Pilz, K.; Fortier, L.A. Hyaluronic Acid Versus Platelet-Rich Plasma: A Prospective, Double-Blind Randomized Controlled Trial Comparing Clinical Outcomes and Effects on Intra-Articular Biology for the Treatment of Knee Osteoarthritis. *Am. J. Sports Med.* **2016**, *45*, 339–346.
12.	(Ong, 2016)	Ong, K.L.; Anderson, A.F.; Niazi, F.; Fierlinger, A.L.; Kurtz, S.M.; Altman, R.D. Hyaluronic Acid Injections in Medicare Knee Osteoarthritis Patients Are Associated With Longer Time to Knee Arthroplasty. *J. Arthroplasty* **2016**, *31*, 1667–1673.
13.	(Paterson, 2016)	Paterson, K.L.; Nicholls, M.; Bennell, K.L.; Bates, D. Intra-Articular Injection of Photo-Activated Platelet-Rich Plasma in Patients with Knee Osteoarthritis: A Double-Blind, Randomized Controlled Pilot Study. *BMC Musculoskelet. Disord.* **2016**, *17*, 67.
14.	(Strand, 2016)	Strand, V.; Lim, S.; Takamura, J. Evidence for Safety of Retreatment with a Single Intra-Articular Injection of Gel-200 for Treatment of Osteoarthritis of the Knee from the Double-Blind Pivotal and Open-Label Retreatment Clinical Trials. *BMC Musculoskelet. Disord.* **2016**, *17*, 240.
15.	(Trojian, 2016)	Trojian, T.H.; Concoff, A.L.; Joy, S.M.; Hatzenbuehler, J.R.; Saulsberry, W.J.; Coleman, C.I. AMSSM Scientific Statement Concerning Viscosupplementation Injections for Knee Osteoarthritis: Importance for Individual Patient Outcomes. *Br. J. Sports Med.* **2016**, *50*, 84–92.
16.	(Vaishya, 2016)	Vaishya, R.; Pariyo, G.B.; Agarwal, A.K.; Vijay, V. Non-Operative Management of Osteoarthritis of the Knee Joint. *J Clin Orthop Trauma* **2016**, *7*, 170–176.
17.	(Temple-Wong, 2016)	Temple-Wong, M.M.; Ren, S.; Quach, P.; Hansen, B.C.; Chen, A.C.; Hasegawa, A.; D'Lima, D.D.; Koziol, J.; Masuda, K.; Lotz, M.K.; et al. Hyaluronan Concentration and Size Distribution in Human Knee Synovial Fluid: Variations with Age and Cartilage Degeneration. *Arthritis Res. Ther.* **2016**, *18*, 18.
18.	(Concoff, 2017)	Concoff, A.; Sancheti, P.; Niazi, F.; Shaw, P.; Rosen, J. The Efficacy of Multiple versus Single Hyaluronic Acid Injections: A Systematic Review and Meta-Analysis. *BMC Musculoskelet. Disord.* **2017**, *18*, 542.

19.	(Cooper, 2017)	Cooper, C.; Rannou, F.; Richette, P.; Bruyère, O.; Al-Daghri, N.; Altman, R.D.; Brandi, M.L.; Collaud Basset, S.; Herrero-Beaumont, G.; Migliore, A.; et al. Use of Intraarticular Hyaluronic Acid in the Management of Knee Osteoarthritis in Clinical Practice. *Arthritis Care Res.* **2017**, *69*, 1287–1296.
20.	(Delbarre, 2017)	Delbarre, A.; Amor, B.; Bardoulat, I.; Tetafort, A.; Pelletier-Fleury, N. Do Intra-Articular Hyaluronic Acid Injections Delay Total Knee Replacement in Patients with Osteoarthritis - A Cox Model Analysis. *PLoS One* **2017**, *12*, e0187227.
21.	(Duymus, 2017)	Duymus, T.M.; Mutlu, S.; Dernek, B.; Komur, B.; Aydogmus, S.; Kesiktas, F.N. Choice of Intra-Articular Injection in Treatment of Knee Osteoarthritis: Platelet-Rich Plasma, Hyaluronic Acid or Ozone Options. *Knee Surg. Sports Traumatol. Arthrosc.* **2017**, *25*, 485–492.
22.	(Görmeli, 2017)	Görmeli, G.; Görmeli, C.A.; Ataoglu, B.; Çolak, C.; Aslantürk, O.; Ertem, K. Multiple PRP Injections Are More Effective than Single Injections and Hyaluronic Acid in Knees with Early Osteoarthritis: A Randomized, Double-Blind, Placebo-Controlled Trial. *Knee Surg. Sports Traumatol. Arthrosc.* **2017**, *25*, 958–965.
23.	(Leung, 2017)	Leung, Y.Y.; Huebner, J.L.; Haaland, B.; Wong, S.B.S.; Kraus, V.B. Synovial Fluid pro-Inflammatory Profile Differs according to the Characteristics of Knee Pain. *Osteoarthritis Cartilage* **2017**, *25*, 1420–1427.
24.	(Raeissadat, 2017)	Raeissadat, S.A.; Rayegani, S.M.; Ahangar, A.G.; Abadi, P.H.; Mojgani, P.; Ahangar, O.G. Efficacy of Intra-Articular Injection of a Newly Developed Plasma Rich in Growth Factor (PRGF) Versus Hyaluronic Acid on Pain and Function of Patients with Knee Osteoarthritis: A Single-Blinded Randomized Clinical Trial. *Clin. Med. Insights Arthritis Musculoskelet. Disord.* **2017**, *10*, 1179544117733452.
25.	(Shewale, 2017)	Shewale, A.R.; Barnes, C.L.; Fischbach, L.A.; Ounpraseuth, S.T.; Painter, J.T.; Martin, B.C. Comparison of Low-, Moderate-, and High-Molecular-Weight Hyaluronic Acid Injections in Delaying Time to Knee Surgery. *J. Arthroplasty* **2017**, *32*, 2952–2957.e21.
26.	(Xing, 2017)	Xing, D.; Wang, B.; Zhang, W.; Yang, Z.; Hou, Y.; Chen, Y.; Lin, J. Intra-Articular Hyaluronic Acid Injection in Treating Knee Osteoarthritis: Assessing Risk of Bias in Systematic Reviews with ROBIS Tool. *Int. J. Rheum. Dis.* **2017**, *20*, 1658–1673.
27.	(Altman, 2018)	Altman, R.; Hackel, J.; Niazi, F.; Shaw, P.; Nicholls, M. Efficacy and Safety of Repeated Courses of Hyaluronic Acid Injections for Knee Osteoarthritis: A Systematic Review. *Semin. Arthritis Rheum.* **2018**, *48*, 168–175.
28.	(Bert, 2018)	Bert, J.; Kenney, J.; Sgaglione, N.A.; McClelland, S.; Brophy, R.; Toth, J.; Ruane, J.; Ali, Y.; Arquette, S.; Dasa, V.; et al. Viscosupplementation for Osteoarthritis of the Knee: A Key Opinion Leader Panel Discussion. *J Manag Care Spec Pharm* **2018**, *24*, S2–S8.
29.	(Bowman, 2018)	Bowman, E.N.; Hallock, J.D.; Throckmorton, T.W.; Azar, F.M. Hyaluronic Acid Injections for Osteoarthritis of the Knee: Predictors of Successful Treatment. *Int. Orthop.* **2018**, *42*, 733–740.
30.	(Cao, 2018)	Cao, Z.; Mai, X.; Wang, J.; Feng, E.; Huang, Y. Unicompartmental Knee Arthroplasty vs High Tibial Osteotomy for Knee

		Osteoarthritis: A Systematic Review and Meta-Analysis. *J. Arthroplasty* **2018**, *33*, 952–959.
31.	(Mora, 2018)	Mora, J.C.; Przkora, R.; Cruz-Almeida, Y. Knee Osteoarthritis: Pathophysiology and Current Treatment Modalities. *J. Pain Res.* **2018**, *11*, 2189–2196.
32.	(Nelson, 2018)	Nelson, F.R.T. The Value of Phenotypes in Knee Osteoarthritis Research. *Open Orthop. J.* **2018**, *12*, 105–114.
33.	(Su, 2018)	Su, K.; Bai, Y.; Wang, J.; Zhang, H.; Liu, H.; Ma, S. Comparison of Hyaluronic Acid and PRP Intra-Articular Injection with Combined Intra-Articular and Intraosseous PRP Injections to Treat Patients with Knee Osteoarthritis. *Clin. Rheumatol.* **2018**, *37*, 1341–1350.
34.	(Bonitz, 2019)	Bonitz, M.; Schaffer, C.; Amling, M.; Poertner, R.; Schinke, T.; Jeschke, A. Secreted Factors from Synovial Fibroblasts Immediately Regulate Gene Expression in Articular Chondrocytes. *Gene* **2019**, *698*, 1–8.
35.	(Delgado, 2019)	Delgado, D.; Garate, A.; Vincent, H.; Bilbao, A.M.; Patel, R.; Fiz, N.; Sampson, S.; Sánchez, M. Current Concepts in Intraosseous Platelet-Rich Plasma Injections for Knee Osteoarthritis. *J Clin Orthop Trauma* **2019**, *10*, 36–41.
36.	(Dhillon, 2019)	Dhillon, M.S.; Patel, S.; Bansal, T. Improvising PRP for Use in Osteoarthritis Knee- Upcoming Trends and Futuristic View. *J Clin Orthop Trauma* **2019**, *10*, 32–35.
37.	(Huang, 2019)	Huang, Y.; Liu, X.; Xu, X.; Liu, J. Intra-Articular Injections of Platelet-Rich Plasma, Hyaluronic Acid or Corticosteroids for Knee Osteoarthritis : A Prospective Randomized Controlled Study. *Orthopade* **2019**, *48*, 239–247.
38.	(Lin, 2019)	Lin, K.-Y.; Yang, C.-C.; Hsu, C.-J.; Yeh, M.-L.; Renn, J.-H. Intra-Articular Injection of Platelet-Rich Plasma Is Superior to Hyaluronic Acid or Saline Solution in the Treatment of Mild to Moderate Knee Osteoarthritis: A Randomized, Double-Blind, Triple-Parallel, Placebo-Controlled Clinical Trial. *Arthroscopy* **2019**, *35*, 106–117.
39.	(Liu, 2019)	Liu, S.-C.; Qiao, X.-F.; Tang, Q.-X.; Li, X.-G.; Yang, J.-H.; Wang, T.-Q.; Xiao, Y.-J.; Qiao, J.-M. Therapeutic Efficacy of Extracorporeal Shock Wave Combined with Hyaluronic Acid on Knee Osteoarthritis. *Medicine* **2019**, *98*, e14589.
40.	(Buendía-López, 2019)	Buendía-López, D.; Medina-Quirós, M.; Fernández-Villacañas Marín, M.Á. Clinical and Radiographic Comparison of a Single LP-PRP Injection, a Single Hyaluronic Acid Injection and Daily NSAID Administration with a 52-Week Follow-up: A Randomized Controlled Trial. *J. Orthop. Traumatol.* **2019**, *19*, 3.
41.	(Maheu, 2019)	Maheu, E.; Bannuru, R.R.; Herrero-Beaumont, G.; Allali, F.; Bard, H.; Migliore, A. Why We Should Definitely Include Intra-Articular Hyaluronic Acid as a Therapeutic Option in the Management of Knee Osteoarthritis: Results of an Extensive Critical Literature Review. *Semin. Arthritis Rheum.* **2019**, *48*, 563–572.
42.	(Matas, 2019)	Matas, J.; Orrego, M.; Amenabar, D.; Infante, C.; Tapia-Limonchi, R.; Cadiz, M.I.; Alcayaga-Miranda, F.; González, P.L.; Muse, E.; Khoury, M.; et al. Umbilical Cord-Derived Mesenchymal Stromal Cells (MSCs) for Knee Osteoarthritis: Repeated MSC Dosing Is Superior to a Single MSC Dose and to Hyaluronic Acid in a

		Controlled Randomized Phase I/II Trial. *Stem Cells Transl. Med.* **2019**, *8*, 215–224.
43.	(López-Ruiz, 2019)	López-Ruiz, E.; Jiménez, G.; Álvarez de Cienfuegos, L.; Antic, C.; Sabata, R.; Marchal, J.A.; Gálvez-Martín, P. Advances of Hyaluronic Acid in Stem Cell Therapy and Tissue Engineering, Including Current Clinical Trials. *Eur. Cell. Mater.* **2019**, *37*, 186–213.
44.	(Tavassoli, 2019)	Tavassoli, M.; Janmohammadi, N.; Hosseini, A.; Khafri, S.; Esmaeilnejad-Ganji, S.M. Single- and Double-Dose of Platelet-Rich Plasma versus Hyaluronic Acid for Treatment of Knee Osteoarthritis: A Randomized Controlled Trial. *World J. Orthop.* **2019**, *10*, 310–326.
45.	(Vaishya, 2019)	Vaishya, R.; Vijay, V.; Lama, P.; Agarwal, A. Does Vitamin D Deficiency Influence the Incidence and Progression of Knee Osteoarthritis? - A Literature Review. *J Clin Orthop Trauma* **2019**, *10*, 9–15.
46.	(Vinod, 2019)	Vinod, E.; James, J.V.; Sabareeswaran, A.; Amirtham, S.M.; Thomas, G.; Sathishkumar, S.; Ozbey, O.; Boopalan, P.R.J.V.C. Intraarticular Injection of Allogenic Chondroprogenitors for Treatment of Osteoarthritis in Rabbit Knee Model. *J Clin Orthop Trauma* **2019**, *10*, 16–23.
47.	(Vitale, 2019)	Vitale, N.D.; Vandenbulcke, F.; Chisari, E.; Iacono, F.; Lovato, L.; Di Matteo, B.; Kon, E. Innovative Regenerative Medicine in the Management of Knee OA: The Role of Autologous Protein Solution. *J Clin Orthop Trauma* **2019**, *10*, 49–52.
48.	(Belsey, 2020)	Belsey, J.; Yasen, S.K.; Jobson, S.; Faulkner, J.; Wilson, A.J. Return to Physical Activity After High Tibial Osteotomy or Unicompartmental Knee Arthroplasty: A Systematic Review and Pooling Data Analysis. *Am. J. Sports Med.* **2020**, *49*, 1372–1380.
49.	(Billesberger, 2020)	Billesberger, L.M.; Fisher, K.M.; Qadri, Y.J.; Boortz-Marx, R.L. Procedural Treatments for Knee Osteoarthritis: A Review of Current Injectable Therapies. *Pain Res. Manag.* **2020**, *2020*, 3873098.
50.	(Chen, 2020)	Chen, L.; Zheng, J.J.Y.; Li, G.; Yuan, J.; Ebert, J.R.; Li, H.; Papadimitrou, J.; Wang, Q.; Wood, D.; Jones, C.W.; Zheng, M. Pathogenesis and clinical management of obesity-related knee osteoarthritis: Impact of mechanical loading. *J Orthop Translat*. **2020**, *24*,66-75.
51.	(Koh, 2020)	Koh, S.M.; Chan, C.K.; Teo, S.H.; Singh, S.; Merican, A.; Ng, W.M.; Abbas, A.; Kamarul, T. Elevated Plasma and Synovial Fluid Interleukin-8 and Interleukin-18 May Be Associated with the Pathogenesis of Knee Osteoarthritis. *Knee* **2020**, *27*, 26–35.
52.	(Long, 2020)	Long, H.; Zeng, X.; Liu, Q.; Wang, H.; Vos, T.; Hou, Y.; Lin, C.; Qiu, Y.; Wang, K.; Xing, D.; et al. Burden of Osteoarthritis in China, 1990–2017: Findings from the Global Burden of Disease Study 2017. *The Lancet Rheumatology* **2020**, *2*, e164–e172.
53.	(Raeissadat, 2020)	Raeissadat, S.A.; Gharooee Ahangar, A.; Rayegani, S.M.; Minator Sajjadi, M.; Ebrahimpour, A.; Yavari, P. Platelet-Rich Plasma-Derived Growth Factor vs Hyaluronic Acid Injection in the Individuals with Knee Osteoarthritis: A One Year Randomized Clinical Trial. *J. Pain Res.* **2020**, *13*, 1699–1711.
54.	(Rezasoltani, 2020)	Rezasoltani, Z.; Azizi, S.; Najafi, S.; Sanati, E.; Dadarkhah, A.; Abdorrazaghi, F. Physical Therapy, Intra-Articular Dextrose

		Prolotherapy, Botulinum Neurotoxin, and Hyaluronic Acid for Knee Osteoarthritis: Randomized Clinical Trial. *Int. J. Rehabil. Res.* **2020**, *43*, 219–227.
55.	(Tang, 2020)	Tang, J.Z.; Nie, M.J.; Zhao, J.Z.; Zhang, G.C.; Zhang, Q.; Wang, B. Platelet-Rich Plasma versus Hyaluronic Acid in the Treatment of Knee Osteoarthritis: A Meta-Analysis. *J. Orthop. Surg. Res.* **2020**, *15*, 403.
56.	(Zhao, 2020)	Zhao, J.; Huang, H.; Liang, G.; Zeng, L.-F.; Yang, W.; Liu, J. Effects and Safety of the Combination of Platelet-Rich Plasma (PRP) and Hyaluronic Acid (HA) in the Treatment of Knee Osteoarthritis: A Systematic Review and Meta-Analysis. *BMC Musculoskelet. Disord.* **2020**, *21*, 224.
57.	(Baria, 2021)	Baria, M.R.; Vasileff, W.K.; Borchers, J.; DiBartola, A.; Flanigan, D.C.; Plunkett, E.; Magnussen, R.A. Treating Knee Osteoarthritis With Platelet-Rich Plasma and Hyaluronic Acid Combination Therapy: A Systematic Review. *Am. J. Sports Med.* **2021**, *50*, 273–281.
58.	(Boffa, 2021)	Boffa, A.; Merli, G.; Andriolo, L.; Lattermann, C.; Salzmann, G.M.; Filardo, G. Synovial Fluid Biomarkers in Knee Osteoarthritis: A Systematic Review and Quantitative Evaluation Using BIPEDs Criteria. *Cartilage* **2021**, *13*, 82S – 103S.
59.	(Belk, 2021)	Belk, J.W.; Kraeutler, M.J.; Houck, D.A.; Goodrich, J.A.; Dragoo, J.L.; McCarty, E.C. Platelet-Rich Plasma Versus Hyaluronic Acid for Knee Osteoarthritis: A Systematic Review and Meta-Analysis of Randomized Controlled Trials. *Am. J. Sports Med.* **2021**, *49*, 249–260.
60.	(Dulic, 2021)	Dulic, O.; Rasovic, P.; Lalic, I.; Kecojevic, V.; Gavrilovic, G.; Abazovic, D.; Maric, D.; Miskulin, M.; Bumbasirevic, M. Bone Marrow Aspirate Concentrate versus Platelet Rich Plasma or Hyaluronic Acid for the Treatment of Knee Osteoarthritis. *Medicina* **2021**, *57*, doi:10.3390/medicina57111193.
61.	(Gupta, 2021)	Gupta, A.; Maffulli, N.; Rodriguez, H.C.; Carson, E.W.; Bascharon, R.A.; Delfino, K.; Levy, H.J.; El-Amin, S.F., 3rd Safety and Efficacy of Umbilical Cord-Derived Wharton's Jelly Compared to Hyaluronic Acid and Saline for Knee Osteoarthritis: Study Protocol for a Randomized, Controlled, Single-Blind, Multi-Center Trial. *J. Orthop. Surg. Res.* **2021**, *16*, 352.
62.	(Karasavvidis, 2021)	Karasavvidis, T.; Totlis, T.; Gilat, R.; Cole, B.J. Platelet-Rich Plasma Combined With Hyaluronic Acid Improves Pain and Function Compared With Hyaluronic Acid Alone in Knee Osteoarthritis: A Systematic Review and Meta-Analysis. *Arthroscopy* **2021**, *37*, 1277–1287.e1.
63.	(Oo, 2021)	Oo, W.M.; Little, C.; Duong, V.; Hunter, D.J. The Development of Disease-Modifying Therapies for Osteoarthritis (DMOADs): The Evidence to Date. *Drug Des. Devel. Ther.* **2021**, *15*, 2921–2945.
64.	(Park, 2021)	Park, Y.-B.; Kim, J.-H.; Ha, C.-W.; Lee, D.-H. Clinical Efficacy of Platelet-Rich Plasma Injection and Its Association With Growth Factors in the Treatment of Mild to Moderate Knee Osteoarthritis: A Randomized Double-Blind Controlled Clinical Trial As Compared With Hyaluronic Acid. *Am. J. Sports Med.* **2021**, *49*, 487–496.

65.	(Raeissadat, 2021)	Raeissadat, S.A.; Ghazi Hosseini, P.; Bahrami, M.H.; Salman Roghani, R.; Fathi, M.; Gharooee Ahangar, A.; Darvish, M. The Comparison Effects of Intra-Articular Injection of Platelet Rich Plasma (PRP), Plasma Rich in Growth Factor (PRGF), Hyaluronic Acid (HA), and Ozone in Knee Osteoarthritis; a One Year Randomized Clinical Trial. *BMC Musculoskelet. Disord.* **2021**, *22*, 134.
66.	(Sdeek, 2021)	Sdeek, M.; Sabry, D.; El-Sdeek, H.; Darweash, A. Intra-Articular Injection of Platelet Rich Plasma versus Hyaluronic Acid for Moderate Knee Osteoarthritis. A Prospective, Double-Blind Randomized Controlled Trial on 189 Patients with Follow-up for Three Years. *Acta Orthop. Belg.* **2021**, *87*, 729–734.
67.	(Tan, 2021)	Tan, J.; Chen, H.; Zhao, L.; Huang, W. Platelet-Rich Plasma Versus Hyaluronic Acid in the Treatment of Knee Osteoarthritis: A Meta-Analysis of 26 Randomized Controlled Trials. *Arthroscopy* **2021**, *37*, 309–325.
68.	(Timur, 2021)	Timur, U.T.; Jahr, H.; Anderson, J.; Green, D.C.; Emans, P.J.; Smagul, A.; van Rhijn, L.W.; Peffers, M.J.; Welting, T.J.M. Identification of Tissue-Dependent Proteins in Knee OA Synovial Fluid. *Osteoarthritis Cartilage* **2021**, *29*, 124–133.
69.	(Wang, 2021)	Wang, S.-J.; Wang, Y.-H.; Huang, L.-C. The Effect of Oral Low Molecular Weight Liquid Hyaluronic Acid Combination with Glucosamine and Chondroitin on Knee Osteoarthritis Patients with Mild Knee Pain: An 8-Week Randomized Double-Blind Placebo-Controlled Trial. *Medicine* **2021**, *100*, e24252.<br>Referinte: No80-No71
70.	(Webner, 2021)	Webner, D.; Huang, Y.; Hummer, C.D., 3rd Intraarticular Hyaluronic Acid Preparations for Knee Osteoarthritis: Are Some Better Than Others? *Cartilage* **2021**, *13*, 1619S – 1636S.
71.	(Zhao, 2021)	Zhao, D.; Pan, J.-K.; Yang, W.-Y.; Han, Y.-H.; Zeng, L.-F.; Liang, G.-H.; Liu, J. Intra-Articular Injections of Platelet-Rich Plasma, Adipose Mesenchymal Stem Cells, and Bone Marrow Mesenchymal Stem Cells Associated With Better Outcomes Than Hyaluronic Acid and Saline in Knee Osteoarthritis: A Systematic Review and Network Meta-Analysis. *Arthroscopy* **2021**, *37*, 2298–2314.e10.
72.	(Bucci, 2022)	Bucci, J.; Chen, X.; LaValley, M.; Nevitt, M.; Torner, J.; Lewis, C.E.; Felson, D.T. Progression of Knee Osteoarthritis With Use of Intraarticular Glucocorticoids Versus Hyaluronic Acid. *Arthritis Rheumatol* **2022**, *74*, 223–226.
73.	(Chavda, 2022)	Chavda, S.; Rabbani, S.A.; Wadhwa, T. Role and Effectiveness of Intra-Articular Injection of Hyaluronic Acid in the Treatment of Knee Osteoarthritis: A Systematic Review. *Cureus* **2022**, *14*, e24503.
74.	(Kim, 2022)	Kim, Y.S.; Guilak, F. Engineering Hyaluronic Acid for the Development of New Treatment Strategies for Osteoarthritis. *Int. J. Mol. Sci.* **2022**, *23*, doi:10.3390/ijms23158662.
75.	(Mao, 2022)	Mao, B.; Pan, Y.; Zhang, Z.; Yu, Z.; Li, J.; Fu, W. Efficacy and Safety of Hyaluronic Acid Intra-Articular Injection after Arthroscopic Knee Surgery: A Systematic Review and Meta-Analysis. *Orthop. Surg.* **2022**, *15*, 16–27.
76.	(Singh, 2022)	Singh, H.; Knapik, D.M.; Polce, E.M.; Eikani, C.K.; Bjornstad, A.H.; Gursoy, S.; Perry, A.K.; Westrick, J.C.; Yanke, A.B.; Verma, N.N.; et al. Relative Efficacy of Intra-Articular Injections in the

		Treatment of Knee Osteoarthritis: A Systematic Review and Network Meta-Analysis. *Am. J. Sports Med.* **2022**, *50*, 3140–3148.
77.	(Ta, 2022)	Ta, C.N.; Vasudevan, R.; Mitchell, B.C.; Keller, R.A.; Kent, W.T. The Influence of Industry Affiliation on Randomized Controlled Trials of Platelet-Rich Plasma for Knee Osteoarthritis. *Am. J. Sports Med.* **2022**, *51*, 3583–3590.
78.	(Wang, 2022)	Wang, Y.-C.; Lee, C.-L.; Chen, Y.-J.; Tien, Y.-C.; Lin, S.-Y.; Chen, C.-H.; Chou, P.P.-H.; Huang, H.-T. Comparing the Efficacy of Intra-Articular Single Platelet-Rich Plasma(PRP) versus Novel Crosslinked Hyaluronic Acid for Early-Stage Knee Osteoarthritis: A Prospective, Double-Blind, Randomized Controlled Trial. *Medicina* **2022**, *58*, doi:10.3390/medicina58081028.
79.	(Zhang, 2022)	Zhang, Q.; Liu, T.; Gu, Y.; Gao, Y.; Ni, J. Efficacy and Safety of Platelet-Rich Plasma Combined with Hyaluronic Acid versus Platelet-Rich Plasma Alone for Knee Osteoarthritis: A Systematic Review and Meta-Analysis. *J. Orthop. Surg. Res.* **2022**, *17*, 499.
80.	(Alahmed, 2023)	Alahmed, S.K.; Mohyeldin, A.M.; Alshammari, A.; Alshammari, Z.F.; Alhamdi, R.A.; Alghaslan, S.A.; Alshammari, H.F.; Alshamry, F.F.; Alshammari, A.H.; Alhamdi, M.S. Knowledge and Awareness Regarding Osteoarthritis and Its Factors in Hail Region, Saudi Arabia. *Cureus* **2023**, *15*, e36557.
81.	(Amirsaadat, 2023)	Amirsaadat, S.; Amirazad, H.; Hashemihesar, R.; Zarghami, N. An Update on the Effect of Intra-Articular Intervention Strategies Using Nanomaterials in Osteoarthritis: Possible Clinical Application. *Front Bioeng Biotechnol* **2023**, *11*, 1128856.
82.	(Andronic, 2023)	Andronic, O.; Claydon-Mueller, L.S.; Cubberley, R.; Karczewski, D.; Lu, V.; Khanduja, V. No Evidence Exists on Outcomes of Non-Operative Management in Patients with Femoroacetabular Impingement and Concomitant Tönnis Grade 2 or More Hip Osteoarthritis: A Scoping Review. *Knee Surg. Sports Traumatol. Arthrosc.* **2023**, *31*, 2103–2122.
83.	(Bąkowski, 2023)	Bąkowski, P.; Kaszyński, J.; Baka, C.; Kaczmarek, T.; Ciemniewska-Gorzela, K.; Bąkowska-Żywicka, K.; Piontek, T. Patients with Stage II of the Knee Osteoarthritis Most Likely Benefit from the Intra-Articular Injections of Autologous Adipose Tissue-from 2 Years of Follow-up Studies. *Arch. Orthop. Trauma. Surg.* **2023**, *143*, 55–62.
84.	(Barman, 2023)	Barman, A.; Bandyopadhyay, D.; Mohakud, S.; Sahoo, J.; Maiti, R.; Mukherjee, S.; Prakash, S.; Roy, S.S.; Viswanath, A. Comparison of Clinical Outcome, Cartilage Turnover, and Inflammatory Activity Following Either Intra-Articular or a Combination of Intra-Articular with Intra-Osseous Platelet-Rich Plasma Injections in Osteoarthritis Knee: A Randomized, Clinical Trial. *Injury* **2023**, *54*, 728–737.
85.	(Belk, 2023)	Belk, J.W.; Lim, J.J.; Keeter, C.; McCulloch, P.C.; Houck, D.A.; McCarty, E.C.; Frank, R.M.; Kraeutler, M.J. Patients With Knee Osteoarthritis Who Receive Platelet-Rich Plasma or Bone Marrow Aspirate Concentrate Injections Have Better Outcomes Than Patients Who Receive Hyaluronic Acid: Systematic Review and Meta-Analysis. *Arthroscopy* **2023**, *39*, 1714–1734.
86.	(Béret, 2023)	Béret, M.; Barry, F.; Garcia-Fernandez, M.-J.; Chijcheapaza-Flores, H.; Blanchemain, N.; Chai, F.; Nicot, R. Efficacy of Intra-Articular Injection of Botulinum Toxin Type A (IncobotulinumtoxinA) in

		Temporomandibular Joint Osteoarthritis: A Three-Arm Controlled Trial in Rats. *Toxins* **2023**, *15*, doi:10.3390/toxins15040261.
87.	(Blicharski, 2023)	Blicharski, T.; Łukasik, P.; Plebanski, R.; Żęgota, Z.; Szuścik, M.; Moster, E.; Pavelka, K.; Jeon, S.; Park, S.L. Efficacy and Safety of Intra-Articular Cross-Linked Sodium Hyaluronate for the Treatment of Knee Osteoarthritis: A Prospective, Active-Controlled, Randomized, Parallel-Group, Double-Blind, Multicenter Study. *J. Clin. Med. Res.* **2023**, *12*, doi:10.3390/jcm12082982.
88.	(Bowden, 2023)	Bowden, D.J.; Eustace, S.J.; Kavanagh, E.C. The Value of Injectable Viscoelastic Supplements for Joints. *Skeletal Radiol.* **2023**, *52*, 933–940.
89.	(Charnwichai, 2023)	Charnwichai, P.; Tammachote, R.; Tammachote, N.; Chaichana, T.; Kitkumthorn, N. Histological Features of Knee Osteoarthritis Treated with Triamcinolone Acetonide and Hyaluronic Acid. *Biomedical Reports* **2023**, *18*, 1–8.
90.	(Chen, 2023)	Chen, L.; Jin, S.; Yao, Y.; He, S.; He, J. Comparison of Clinical Efficiency between Intra-Articular Injection of Platelet-Rich Plasma and Hyaluronic Acid for Osteoarthritis: A Meta-Analysis of Randomized Controlled Trials. *Ther. Adv. Musculoskelet. Dis.* **2023**, *15*, 1759720X231157043.
91.	(Costa, 2023)	Costa, L.A.V.; Lenza, M.; Irrgang, J.J.; Fu, F.H.; Ferretti, M. How Does Platelet-Rich Plasma Compare Clinically to Other Therapies in the Treatment of Knee Osteoarthritis? A Systematic Review and Meta-Analysis. *Am. J. Sports Med.* **2023**, *51*, 1074–1086.
92.	(Conrozier, 2023)	Conrozier, T.; Diraçoglù, D.; Monfort, J.; Chevalier, X.; Bard, H.; Baron, D.; Jerosch, J.; Migliore, A.; Richette, P.; Henrotin, Y. EUROVISCO Good Practice Recommendations for a First Viscosupplementation in Patients with Knee Osteoarthritis. *Cartilage* **2023**, *14*, 125–135.
93.	(Clausen, 2023)	Clausen, S.; Heerey, J.; Hartvigsen, J.; Kemp, J.L.; Arnbak, B. Do Imaging Findings Modify the Effect of Non-Surgical Treatment in Patients with Knee and Hip Osteoarthritis? A Systematic Literature Review. *BMJ Open* **2023**, *13*, e065373.
94.	(Ciapini, 2023)	Ciapini, G.; Simonettii, M.; Giuntoli, M.; Varchetta, G.; De Franco, S.; Ipponi, E.; Scaglione, M.; Parchi, P.D. Is the Combination of Platelet-Rich Plasma and Hyaluronic Acid the Best Injective Treatment for Grade II-III Knee Osteoarthritis? A Prospective Study. *Adv. Orthop.* **2023**, *2023*, 1868943.
95.	(Chopin, 2023)	Chopin, C.; Geoffroy, M.; Kanagaratnam, L.; Dorilleau, C.; Ecarnot, F.; Siboni, R.; Salmon, J.-H. Prognostic Factors Related to Clinical Response in 210 Knees Treated by Platelet-Rich Plasma for Osteoarthritis. *Diagnostics (Basel)* **2023**, *13*, doi:10.3390/diagnostics13040760.
96.	(Cui, 2023)	Cui, Y.; Lin, L.; Wang, Z.; Wang, K.; Xiao, L.; Lin, W.; Zhang, Y. Research Trends of Platelet-Rich Plasma Therapy on Knee Osteoarthritis from 2011 to 2021: A Review. *Medicine* **2023**, *102*, e32434.
97.	(Gezer, 2023)	Gezer, H.H.; Ostor, A. What Is New in Pharmacological Treatment for Osteoarthritis? *Best Pract. Res. Clin. Rheumatol.* **2023**, *37*, 101841.
98.	(Gomes, 2023)	Gomes, F.F.; Maranho, D.A.; Gomes, M.S.; de Castro, I.M., Jr; Mansur, H. Effects of Hyaluronic Acid With Intra-Articular

		Corticosteroid Injections in the Management of Subtalar Post-Traumatic Osteoarthritis - Randomized Comparative Trial. *J. Foot Ankle Surg*. **2023**, *62*, 14–20.
99.	(Gomoll, 2023)	Gomoll, A.H.; Mandelbaum, B.R.; Farr, J.; Archambault, W.T.; Sherman, S.L.; Tabet, S.K.; Kimmerling, K.A.; Mowry, K.C. An Initial Injection and a Crossover Injection of Amniotic Suspension Allograft Following Failed Treatment with Hyaluronic Acid or Saline Are Equally Effective in the Treatment of Moderate Symptomatic Knee Osteoarthritis Over 12 Months. *Arthroscopy* **2023**, *39*, 66–78.
100.	(Habibi, 2023)	Habibi, A.A.; Karia, R.J.; Ward, S.A.; Schwarzkopf, R.; Rozell, J.C.; Slover, J.D. Patient-Reported Outcomes Following Intra-Articular Hyaluronic Acid for Knee Osteoarthritis. *J. Arthroplasty* **2023**, *38*, S36–S41.
101.	(Hegab, 2023)	Hegab, A.F.; Hameed, H.I.A.A.; Hassaneen, A.M.; Hyder, A. Synergistic Effect of Platelet Rich Plasma with Hyaluronic Acid Injection Following Arthrocentesis to Reduce Pain and Improve Function in TMJ Osteoarthritis. *J Stomatol Oral Maxillofac Surg* **2023**, *124*, 101340.
102.	(Householder, 2023)	Householder, N.A.; Raghuram, A.; Agyare, K.; Thipaphay, S.; Zumwalt, M. A Review of Recent Innovations in Cartilage Regeneration Strategies for the Treatment of Primary Osteoarthritis of the Knee: Intra-Articular Injections. *Orthop J Sports Med* **2023**, *11*, 23259671231155950.
103.	(Jang, 2023)	Jang, C.W.; Bang, M.; Park, J.H.; Cho, H.E. Impact of Changes in Clinical Practice Guidelines for Intra-Articular Injection Treatments for Knee Osteoarthritis on Public Interest and Social Media. *Osteoarthritis Cartilage* **2023**, *31*, 793–801.
104.	(Kamat, 2023)	Kamat, Y.D.; Das, B.; Thakkar, K.; Mahajan, M. A Retrospective Observational Study Evaluating the Synergistic Effect of a Novel Combination of Alfapin + Native Type 2 Collagen + Mobilee (Hyaluronic Acid) + CurQlife (Curcumin) Nutraceuticals in the Symptomatic Improvement of Knee Osteoarthritis. *Cureus* **2023**, *15*, e36123.
105.	(Khan, 2023)	Khan, M.; Shanmugaraj, A.; Prada, C.; Patel, A.; Babins, E.; Bhandari, M. The Role of Hyaluronic Acid for Soft Tissue Indications: A Systematic Review and Meta-Analysis. *Sports Health* **2023**, *15*, 86–96.
106.	(Kim, 2023)	Kim, T.W.; Chang, M.J.; Shin, C.Y.; Chang, C.B.; Kang, S.-B. A Randomized Controlled Trial for Comparing Efficacy and Safety between Intraarticular Polynucleotide and Hyaluronic Acid for Knee Osteoarthritis Treatment. *Sci. Rep.* **2023**, *13*, 9419.
107.	(Lambova, 2023)	Lambova, S.N. Knee Osteoarthritis-How Close Are We to Disease-Modifying Treatment: Emphasis on Metabolic Type Knee Osteoarthritis. *Life* **2023**, *13*, doi:10.3390/life13010140.
108.	(Li, 2023)	Li, S.; Xing, F.; Yan, T.; Zhang, S.; Chen, F. Multiple Injections of Platelet-Rich Plasma Versus Hyaluronic Acid for Knee Osteoarthritis: A Systematic Review and Meta-Analysis of Current Evidence in Randomized Controlled Trials. *J Pers Med* **2023**, *13*, doi:10.3390/jpm13030429.
109.	(Liao, 2023)	Liao, C.-D.; Chen, H.-C.; Huang, M.-H.; Liou, T.-H.; Lin, C.-L.; Huang, S.-W. Comparative Efficacy of Intra-Articular Injection, Physical Therapy, and Combined Treatments on Pain, Function,

		and Sarcopenia Indices in Knee Osteoarthritis: A Network Meta-Analysis of Randomized Controlled Trials. *Int. J. Mol. Sci.* **2023**, *24*, doi:10.3390/ijms24076078.
110.	(Lu, 2023)	Lu, K.-H.; Lu, P.W.-A.; Lin, C.-W.; Lu, E.W.-H.; Yang, S.-F. Different Molecular Weights of Hyaluronan Research in Knee Osteoarthritis: A State-of-the-Art Review. *Matrix Biol.* **2023**, *117*, 46–71.
111.	(Luo, 2023)	Luo, Y.; Tan, J.; Zhou, Y.; Guo, Y.; Liao, X.; He, L.; Li, D.; Li, X.; Liu, Y. From Crosslinking Strategies to Biomedical Applications of Hyaluronic Acid-Based Hydrogels: A Review. *Int. J. Biol. Macromol.* **2023**, *231*, 123308.
112.	(Mai, 2023)	Mai, Y.; Zhang, J.; Huang, G.; He, J.; Liu, X.; Guo, L.; Wei, Z.; Jiang, L. Efficacy of the Combination Therapy of Platelet-Rich Plasma and Hyaluronic Acid on Improving Knee Pain and Dysfunction in Patients with Moderate-to-Severe KOA: A Protocol for a Randomised Controlled Trial. *BMJ Open* **2023**, *13*, e068743.
113.	(Molloy, 2023)	Molloy, I.B.; Holte, A.J.; Zhao, Y.; Parker, D.J.; Werth, P.M.; Jevsevar, D.S. The Effect of Intra-Articular Hyaluronic Acid Injections and Payer Coverage on Total Knee Arthroplasty Procedures: Evidence From Large US Claims Database. *Arthroplast Today* **2023**, *19*, 101080.
114.	(Moon, 2023)	Moon, J.Y.; Kim, J.; Lee, J.Y.; Ko, Y.; Park, H.J.; Jeon, Y.H. Comparison of Polynucleotide, Sodium Hyaluronate, and Crosslinked Sodium Hyaluronate for the Management of Painful Knee Osteoarthritis: A Multi-Center, Randomized, Double-Blind, Parallel-Group Study. *Pain Med.* **2023**, *24*, 496–506.
115.	(Muñoz-Salamanca, 2023)	Muñoz-Salamanca, J.A.; Gutierrez, M.; Echevarría-Trujillo, Á. Retrograde "Sandwich" Technique and Implantation of Minced Cartilage in a Hyaluronic Acid Scaffold for Deep Osteochondral Knee Lesions. *Arthrosc Tech* **2023**, *12*, e395–e400.
116.	(Perruchet, 2023)	Perruchet, S.; Balblanc, J.-C.; Rapp, C.; Bourgoin, C.; Guillochon, C.; Lohse, A.; Conrozier, T. The Association between Radiographic Features and the Duration of Effectiveness of a Single Injection of Extended-Release Hyaluronic Acid (HANOX-M-XL) in Patients with Knee Osteoarthritis: Preliminary Results of a Prospective Trial. *Cartilage* **2023**, *14*, 136–143.
117.	(Porcello, 2023)	Porcello, A.; Gonzalez-Fernandez, P.; Jeannerat, A.; Peneveyre, C.; Abdel-Sayed, P.; Scaletta, C.; Raffoul, W.; Hirt-Burri, N.; Applegate, L.A.; Allémann, E.; et al. Thermo-Responsive Hyaluronan-Based Hydrogels Combined with Allogeneic Cytotherapeutics for the Treatment of Osteoarthritis. *Pharmaceutics* **2023**, *15*, 1528.
118.	(Raeissadat, 2023)	Raeissadat, S.A.; Rahimi, M.; Rayegani, S.M.; Moradi, N. Cost-Utility Analysis and Net Monetary Benefit of Platelet Rich Plasma (PRP), Intra-Articular Injections in Compared to Plasma Rich in Growth Factors (PRGF), Hyaluronic Acid (HA) and Ozone in Knee Osteoarthritis in Iran. *BMC Musculoskelet. Disord.* **2023**, *24*, 22.
119.	(Richard, 2023)	Richard, M.J.; Driban, J.B.; McAlindon, T.E. Pharmaceutical Treatment of Osteoarthritis. *Osteoarthritis Cartilage* **2023**, *31*, 458–466.
120.	(Sahin, 2023)	Sahin, N.; Yesil, H. Regenerative Methods in Osteoarthritis. *Best Pract. Res. Clin. Rheumatol.* **2023**, *37*, 101824.

121.	(Saiz, 2023)	Saiz, L.C.; Erviti, J.; Leache, L.; Gutiérrez-Valencia, M. Restoring Study PRGF: A Randomized Clinical Trial on Plasma Rich in Growth Factors for Knee Osteoarthritis. *Trials* **2023**, *24*, 37.
122.	(Saviola, 2023)	Saviola, G.; Da Campo, G.; Bianchini, M.C.; Abdi-Ali, L.; Comini, L.; Rosini, S.; Molfetta, L. Intra-Articular Clodronate in Patients with Knee Osteoarthritis Non-Responder to Intra-Articular Hyaluronic Acid - a Case Report Series of 9 Patients with 8-Month Follow-Up. *Clin. Ter.* **2023**, *174*, 245–248.
123.	(Sconza, 2023)-1	Sconza, C.; Di Matteo, B.; Queirazza, P.; Dina, A.; Amenta, R.; Respizzi, S.; Massazza, G.; Ammendolia, A.; Kon, E.; de Sire, A. Ozone Therapy versus Hyaluronic Acid Injections for Pain Relief in Patients with Knee Osteoarthritis: Preliminary Findings on Molecular and Clinical Outcomes from a Randomized Controlled Trial. *Int. J. Mol. Sci.* **2023**, *24*, doi:10.3390/ijms24108788.
124.	(Sconza, 2023)-2	Sconza, C.; Leonardi, G.; Carfì, C.; Kon, E.; Respizzi, S.; Scaturro, D.; Letizia Mauro, G.; Massazza, G.; Di Matteo, B. Intra-Articular Injection of Botulinum Toxin for the Treatment of Knee Osteoarthritis: A Systematic Review of Randomized Controlled Trials. *Int. J. Mol. Sci.* **2023**, *24*, doi:10.3390/ijms24021486.
125.	(Shen, 2023)	Shen, J.; Lin, X.; Lin, Y.; Xiao, J.; Wu, C.; Zheng, F.; Wu, X.; Lin, H.; Chen, G.; Liu, H. Supplementation of Hyaluronic Acid Injections with Vitamin D Improve Knee Function by Attenuating Synovial Fluid Oxidative Stress in Osteoarthritis Patients with Vitamin D Insufficiency. *Front Nutr* **2023**, *10*, 1026722.
126.	(Simental-Mendía, 2023)	Simental-Mendía, M.; Ortega-Mata, D.; Tamez-Mata, Y.; Olivo, C.A.A.; Vilchez-Cavazos, F. Comparison of the Clinical Effectiveness of Activated and Non-Activated Platelet-Rich Plasma in the Treatment of Knee Osteoarthritis: A Systematic Review and Meta-Analysis. *Clin. Rheumatol.* **2023**, *42*, 1397–1408.
127.	(Siren, 2023)	Siren, J.; Rämö, L.; Rantasalo, M.; Komulainen, O.; Skants, N.; Reito, A.; Kosola, J.; Lindahl, J. Unicompartmental knee arthroplasty vs. high tibial osteotomy for medial knee osteoarthritis (UNIKORN): a study protocol of a randomized controlled trial. *Trials* **2023**, *24(1)*, 256.
128.	(Srinivasan, 2023)	Srinivasan, V.; Ethiraj, P.; Agarawal, S.; H S, A.; Parmanantham, M. Comparison of Various Modalities in the Treatment of Early Knee Osteoarthritis: An Unsolved Controversy. *Cureus* **2023**, *15*, e33630.
129.	(Tarantino, 2023)	Tarantino, D.; Mottola, R.; Palermi, S.; Sirico, F.; Corrado, B.; Gnasso, R. Intra-Articular Collagen Injections for Osteoarthritis: A Narrative Review. *Int. J. Environ. Res. Public Health* **2023**, *20*, doi:10.3390/ijerph20054390.
130.	(Tschopp, 2023)	Tschopp, M.; Pfirrmann, C.W.A.; Fucentese, S.F.; Brunner, F.; Catanzaro, S.; Kühne, N.; Zwyssig, I.; Sutter, R.; Götschi, T.; Tanadini, M.; et al. A Randomized Trial of Intra-Articular Injection Therapy for Knee Osteoarthritis. *Invest. Radiol.* **2023**, *58*, 355–362.
131.	(Utamawatin, 2023)	Utamawatin, K.; Phruetthiphat, O.-A.; Apinyankul, R.; Chaiamnuay, S. The Efficacy of Intra-Articular Triamcinolone Acetonide 10 Mg vs. 40 Mg in Patients with Knee Osteoarthritis: A Non-Inferiority, Randomized, Controlled, Double-Blind, Multicenter Study. *BMC Musculoskelet. Disord.* **2023**, *24*, 92.
132.	(Vilchez-Cavazos, 2023)	Vilchez-Cavazos, F.; Blázquez-Saldaña, J.; Gamboa-Alonso, A.A.; Peña-Martínez, V.M.; Acosta-Olivo, C.A.; Sánchez-García, A.;

		Simental-Mendía, M. The Use of Platelet-Rich Plasma in Studies with Early Knee Osteoarthritis versus Advanced Stages of the Disease: A Systematic Review and Meta-Analysis of 31 Randomized Clinical Trials. *Arch. Orthop. Trauma. Surg.* **2023**, *143*, 1393–1408.
133.	(Vinet, 2023)	Vinet, M.; Le Stum, M.; Gicquel, T.; Clave, A.; Dubrana, F. Unicompartmental Knee Arthroplasty: A French Multicenteric Retrospective Descriptive Study from 2009 to 2019 with Projections to 2050. *Orthop. Traumatol. Surg. Res.* **2023**, *109*, 103581.
134.	(Wang, 2023)	Wang, P.-H.; Wu, C.-H.; Ma, C.-H.; Chiu, Y.-C.; Wu, P.-T.; Jou, I.-M. Comparison of Intra-Articular Injection of ArtiAid®-Mini with Ostenil®-Mini for Trapeziometacarpal Osteoarthritis: A Double-Blind, Prospective, Randomized, Non-Inferiority Trial. *Jt Dis Relat Surg* **2023**, *34*, 50–57.
135.	(Xie, 2023)	Xie, G.; Wang, T.; Zhou, H.; Yi, L.; He, J.; Zhang, J.; Li, M.; Yang, Y. Repairing Effect and Mechanism of Hydroxysafflor Yellow A and Sodium Hyaluronate for Knee Osteoarthritis in Rabbits. *Altern. Ther. Health Med.* **2023**, *29*, 146–151.
136.	(Xue, 2023)	Xue, Y.; Wang, X.; Wang, X.; Huang, L.; Yao, A.; Xue, Y. A Comparative Study of the Efficacy of Intra-Articular Injection of Different Drugs in the Treatment of Mild to Moderate Knee Osteoarthritis: A Network Meta-Analysis. *Medicine* **2023**, *102*, e33339.
137.	(Ying, 2023)	Ying, H.; Shen, C.; Pan, R.; Li, X.; Chen, Y. Strategy Insight: Mechanical Properties of Biomaterials' Influence on Hydrogel-Mesenchymal Stromal Cell Combination for Osteoarthritis Therapy. *Front. Pharmacol.* **2023**, *14*, 1152612.
138.	(Zhang, 2023)	Zhang, Y.; Ruan, G.; Zheng, P.; Huang, S.; Zhou, X.; Liu, X.; Hu, W.; Feng, H.; Lin, Y.; He, J.; et al. Efficacy and Safety of GLucocorticoid Injections into InfrapaTellar faT Pad in Patients with Knee ostEoarthRitiS: Protocol for the GLITTERS Randomized Controlled Trial. *Trials* **2023**, *24*, 6.
139.	(Zhou, 2023)-1	Zhou, K.; Li, Y.-J.; Soderblom, E.J.; Reed, A.; Jain, V.; Sun, S.; Moseley, M.A.; Kraus, V.B. A "Best-in-Class" Systemic Biomarker Predictor of Clinically Relevant Knee Osteoarthritis Structural and Pain Progression. *Sci Adv* **2023**, *9*, eabq5095.
140.	(Zhou, 2023)-2	Zhou, Y.; Li, H.; Cao, S.; Han, Y.; Shao, J.; Fu, Q.; Wang, B.; Wu, J.; Xiang, D.; Liu, Z.; et al. Clinical Efficacy of Intra-Articular Injection with P-PRP Versus that of L-PRP in Treating Knee Cartilage Lesion: A Randomized Controlled Trial. *Orthop. Surg.* **2023**, *15*, 740–749.
